# High‐Resolution PM_2.5_ Mortality Projections With Machine Learning Reveal Global Inequities Under Future Climate and Socioeconomic Pathways

**DOI:** 10.1029/2026GH001829

**Published:** 2026-11-10

**Authors:** Hemraj Bhattarai, Maria Val Martin, Xueying Liu, Jia Mao, Amos P. K. Tai

**Affiliations:** ^1^ Department of Earth and Environmental Sciences The Chinese University of Hong Kong Hong Kong China; ^2^ Division of Environment and Sustainability The Hong Kong University of Science and Technology Hong Kong China; ^3^ Leverhulme Centre for Climate Change Mitigation School of Biosciences University of Sheffield Sheffield UK; ^4^ Now at Research Institute of Water and Environmental Engineering (IIAMA) Universidad Politécnica de Valencia Valencia Spain; ^5^ Now at Valencian Foundation for Excellence in Research (ValER) Castelló de la Plana Spain; ^6^ Faculty of Applied Sciences Macao Polytechnic University Macao China; ^7^ State Key Laboratory of Agrobiotechnology and Institute of Environment Energy and Sustainability The Chinese University of Hong Kong Hong Kong China

**Keywords:** PM_2.5_‐attributable mortality, machine learning, shared socioeconomic pathways (SSPs), climate‐air quality interactions, global health disparities, air pollution

## Abstract

Fine particulate matter (PM_2.5_) remains a leading environmental risk to global health, yet projections of future PM_2.5_‐attributable mortality remain uncertain because of coarse model resolution and difficulty disentangling the effects of emissions, climate change, and land use. Here, we present a high‐resolution assessment of present‐day (2010) and future (2050) PM_2.5_‐related mortality by integrating Earth system modeling, machine learning, and epidemiological analysis within a factorial forcing framework. We apply an Extreme Gradient Boosting model to bias‐correct and spatially refine Community Earth System Model simulations to 0.1° × 0.1° resolution. Using the Global Exposure Mortality Model, we estimate age‐ and cause‐specific mortality under four Shared Socioeconomic Pathways (SSP1, SSP2, SSP3, SSP5), explicitly isolating the contributions of emissions, climate change, and land use change. We estimate a global PM_2.5_‐attributable mortality burden of approximately 6.13 (95% uncertainty interval: 5.77–6.48) million deaths in 2010. Future mortality trajectories diverge sharply across SSPs: the sustainable pathway (SSP1) reduces global mortality by approximately 1.1 million deaths by 2050, whereas the regional rivalry pathway (SSP3) increases mortality by approximately 0.5 million deaths. Although emissions remain the dominant driver, climate change consistently offsets parts of health benefits achieved through emission reductions. Mortality impacts are concentrated among older populations (≥60 years) and disproportionately affect low‐ and middle‐income countries. A complementary analysis using SSP‐specific population projections demonstrates that future demographic change can substantially modify the projected mortality burden. Our findings underscore the need for integrated emission mitigation, climate action, and public health adaptation to achieve equitable and sustained reductions in PM_2.5_‐related mortality.

## Plain Language Summary

Air pollution from fine particulate matter (PM_2.5_) is one of the leading environmental causes of premature death worldwide. Predicting future health impacts remains challenging because air pollution is influenced by emissions, climate change, land‐use change, and population trends. In this study, we combined an Earth system model with machine learning to produce high‐resolution global PM_2.5_ maps and estimate premature deaths under four future socioeconomic pathways for 2050. We estimated that PM_2.5_ contributed to approximately 6.13 million premature deaths globally in 2010. Future health outcomes differ substantially depending on socioeconomic development. A sustainable pathway with strong emission controls could reduce annual deaths by about 1.1 million, whereas a pathway characterized by weak environmental governance could increase deaths by about 0.5 million. Emission changes are the largest driver of future mortality, but climate change consistently offsets part of the health benefits achieved through cleaner air. Older adults and people living in low‐ and middle‐income countries experience the greatest health burden. A complementary analysis using future population projections also shows that demographic change can substantially modify future mortality estimates. These findings highlight the need for coordinated air quality, climate, and public health policies to reduce the global burden of PM_2.5_ exposure.

## Key Points

- High‐resolution modeling reveals global PM_2.5_ mortality burdens and separates impacts of emissions, climate, and land‐use changes
- Emission reductions lower future deaths, but climate change offsets health gains across socioeconomic pathways
- PM_2.5_‐induced mortality remains concentrated in older populations and low‐ to middle‐income countries, highlighting equity challenges

## 1 Introduction

Fine particulate matter (PM_2.5_) is widely recognized as one of the most consequential environmental risk factors for human health, contributing to millions of premature deaths each year and imposing a substantial burden on public health systems, particularly in low‐ and middle‐income countries (Apte et al., 2015; Bu et al., 2021; Burnett et al., 2018; Sang et al., 2022). A large body of epidemiological and modeling evidence has established robust associations between both short‐ and long‐term PM_2.5_ exposure and increased mortality from cardiovascular, respiratory, and metabolic diseases, as well as lung cancer, across diverse populations and exposure levels (Di et al., 2017; Hayes et al., 2020; Lelieveld et al., 2015; Pope et al., 2019; Shaddick et al., 2018; Yu et al., 2024). Importantly, recent studies demonstrate that adverse health effects occur even at PM_2.5_ concentrations below current World Health Organization (WHO) and national air quality standards, highlighting the inadequacy of existing thresholds and the urgency of further mitigation (Di et al., 2017; Hayes et al., 2020; Shi et al., 2016; Weichenthal et al., 2022).

Global assessments of PM_2.5_‐attributable mortality have been central to understanding the scale of this public health challenge. The Global Burden of Disease (GBD) project estimates that ambient PM_2.5_ exposure is responsible for over 4 million deaths annually, with the heaviest burdens concentrated in rapidly industrializing regions of Asia and Africa (Cohen et al., 2017; Sang et al., 2022). In 2019, air pollution ranked as the fourth leading global risk factor, contributing to approximately 6.7 million deaths worldwide (Murray et al., 2020). However, substantial discrepancies remain among mortality estimates. For example, while the Integrated Exposure‐Response (IER) framework attributes 4.2 million deaths to PM_2.5_ in 2015 (Cohen et al., 2017), the Global Exposure Mortality Model (GEMM) estimates nearly double that burden (8.9 million deaths) for the same year (Burnett et al., 2018). These differences primarily arise from fundamental contrasts in model construction: the IER integrates risks across multiple exposure sources–including household air pollution, second‐hand smoke, and active smoking–to extrapolate risks at high concentrations, whereas GEMM relies on cohort evidence from ambient PM_2.5_ exposure alone and incorporates updated epidemiological data and broader mortality endpoints. In addition, the two models employ different exposure‐response functions and underlying data sets, which further contribute to divergence in estimated risks. Differences in PM_2.5_ spatial resolution or atmospheric model biases, while relevant for exposure assessment, are not the only drivers of disagreement between the two frameworks. Nevertheless, coarse, country‐level or regional averages can obscure pollution hotspots and misrepresent exposure in densely populated areas, particularly across South and East Asia (Pozzer et al., 2023).

The health impacts of PM_2.5_ span multiple disease categories, including ischemic heart disease (IHD), stroke, chronic obstructive pulmonary disease (COPD), lung cancer, and lower respiratory infections (LRI) (Burnett et al., 2018; Lelieveld et al., 2015; Mainka & Żak, 2022; Manisalidis et al., 2020; Yu et al., 2024). Vulnerable groups—such as children, the elderly, and socioeconomically disadvantaged populations—experience disproportionately higher risks due to elevated exposure, pre‐existing health conditions, and limited access to healthcare. Despite decades of air quality regulation, exposure remains nearly ubiquitous: in 2019, more than 90% of the global population lived in areas exceeding the 2005 WHO annual PM_2.5_ guideline of 10 μg m^−3^ (WHO: WHO global air quality guidelines, 2021). Following the recent tightening of the guideline to 5 μg m^−3^, only 1.21% of the world population now resides in regions meeting this standard (Xu et al., 2025), underscoring the unprecedented scale of global PM_2.5_ exposure.

Looking forward, future PM_2.5_ concentrations—and their associated health burdens—are highly uncertain and strongly dependent on socioeconomic development pathways as described by the Shared Socioeconomic Pathways (SSPs) (Bhattarai et al., 2024b; O'Neill et al., 2014). SSPs are scenario families that describe socioeconomic development and are commonly paired with radiative forcing pathways (e.g., SSP1‐2.6, SSP3‐7.0). Anthropogenic emissions remain the dominant driver of PM_2.5_ in many regions, particularly under the regional rivalry pathway (SSP3), where rapid population growth and weak environmental regulations are projected to exacerbate air pollution in developing regions of Asia (Bhattarai et al., 2024b). At the same time, climate change is expected to intensify natural PM_2.5_ sources, including wildfires in the northern extratropics, potentially offsetting gains from emission controls (Bhattarai et al., 2025). Even under sustainable development scenarios (SSP1), reductions in PM_2.5_ exposure may not translate into proportional mortality declines due to demographic changes such as population growth (Yang et al., 2023). Moreover, studies suggest that even in an extreme scenario with zero anthropogenic emissions, more than half of the global population would still be exposed to PM_2.5_ above health‐based guidelines, driven by natural sources such as dust and biomass burning (Pai et al., 2022).

Together, these findings indicate that future PM_2.5_‐related mortality will emerge from complex interactions among emissions trajectories, land use change, climate variability, and demographic dynamics (Chen et al., 2023; Clark et al., 2024; Park et al., 2024; Yang et al., 2023). Land‐use and land‐cover change influences PM_2.5_ by modifying land surface properties that regulate dust emissions, biogenic volatile organic compound (BVOC) emissions, and dry deposition, whereas climate change affects atmospheric chemistry, aerosol formation, precipitation scavenging, atmospheric stagnation, and natural emissions (Bhattarai et al., 2024b; Val Martin et al., 2015). However, most existing scenario‐based assessments rely on coarse‐resolution chemical transport models and focus on total PM_2.5_ or aggregate mortality outcomes. These approaches rarely disentangle the relative contributions of individual forcing factors such as land use, climate change, and emissions. As a result, the spatial heterogeneity of exposure, the dominance of specific drivers in different regions, and the implications for targeted mitigation strategies remain insufficiently understood.

Several critical research gaps therefore persist. First, there is a lack of high‐resolution, global estimates of PM_2.5_‐attributable mortality that simultaneously account for the independent and combined effects of land use change, climate change, and anthropogenic and biomass burning emissions under multiple SSPs (McDuffie et al., 2021; Sang et al., 2022). Second, uncertainties in model‐derived PM_2.5_—stemming from biases in emissions inventories, chemical mechanisms, and meteorology—remain particularly large in regions with sparse monitoring networks, and most global assessments have not fully leveraged recent advances in machine learning (ML) for bias correction and spatial downscaling (Chowdhury et al., 2018; Fang et al., 2016; Silva et al., 2016, 2017). Third, comprehensive, age‐ and cause‐specific mortality projections at high spatial resolution are still rare, limiting the ability to identify vulnerable populations and design targeted public health interventions (Bowe et al., 2019; Chen & Hoek, 2020; Yang et al., 2020, 2021). To the best of our knowledge, no previous study has simultaneously (a) isolated the individual contributions of LULCC, climate, and emissions to future PM_2.5_‐attributable mortality within a consistent Earth system model framework; (b) applied machine learning (ML)‐based statistical bias correction to generate high‐resolution (0.1°) PM_2.5_ fields for multiple SSPs; and (c) quantified age‐specific, cause‐specific, and income‐based inequalities in future mortality.

To address these gaps, this study presents a novel, multi‐dimensional assessment of present‐day (2010) and future (2050) PM_2.5_‐related mortality by integrating advanced Earth system modeling, ML, and health impact assessment. First, using a factorial experiment framework with the Community Earth System Model (CESM) simulations, we systematically isolate and quantify the individual and combined effects of land use change, climate change, and emissions on PM_2.5_ exposure under four SSPs (SSP1, SSP2, SSP3, SSP5), enabling explicit attribution of dominant health risk drivers across regions and socioeconomic pathways. Second, we apply an Extreme Gradient Boosting (XGBoost) ML model to bias‐correct and statistically refine CESM PM_2.5_ fields to a high spatial resolution (0.1° × 0.1°), substantially improving exposure representation and capturing subgrid pollution gradients that are critical for health impact assessment, particularly in data‐sparse regions. Third, we leverage these refined exposure estimates to generate global, high‐resolution, age‐ and cause‐specific mortality estimates for both present day and future. This allows us to identify not only geographic hotspots but also the most vulnerable populations and disease endpoints under each scenario. By synthesizing this factorial forcing analysis, ML‐refined exposure fields, and detailed health impact quantification, this work delivers a comprehensive, policy‐relevant toolkit to disentangle the complex interplay of environmental and demographic drivers of PM_2.5_ mortality. Our findings provide essential insights for designing targeted, scenario‐specific interventions to mitigate the future global health burden of air pollution.

## 2 Methodology

### 2.1 Integrated Framework

This study integrates Earth system modeling, ML, and epidemiological modeling (GEMM) to project PM_2.5_‐induced mortality under future SSP scenarios (Figure 1). The approach consists of three primary steps: (a) simulation of PM_2.5_ and related atmospheric variables using CESM, (b) ML‐based bias correction and spatial refinement using the XGBoost algorithm, and (c) health impact assessment using GEMM. The simulated PM_2.5_, atmospheric constituents, and meteorological variables at the native CESM resolution (0.94° × 1.25°) are regridded to a 0.1° × 0.1° resolution via bilinear interpolation to spatially align with the satellite‐derived observational PM_2.5_ data (version 5) from Van Donkelaar et al. (2021). These aligned data sets are then processed using the ML model to obtain bias‐corrected and refined PM_2.5_ estimates. The corrected PM_2.5_ fields are integrated with gridded population density and baseline mortality rates for non‐communicable diseases and LRI (NCD + LRI). Using GEMM, cause‐ and age‐specific mortality burdens are estimated for each scenario, encompassing various forcing types (land use, climate, emissions, and their total combined effect) across four SSPs (SSP1, SSP2, SSP3, and SSP5).

**Figure 1:**
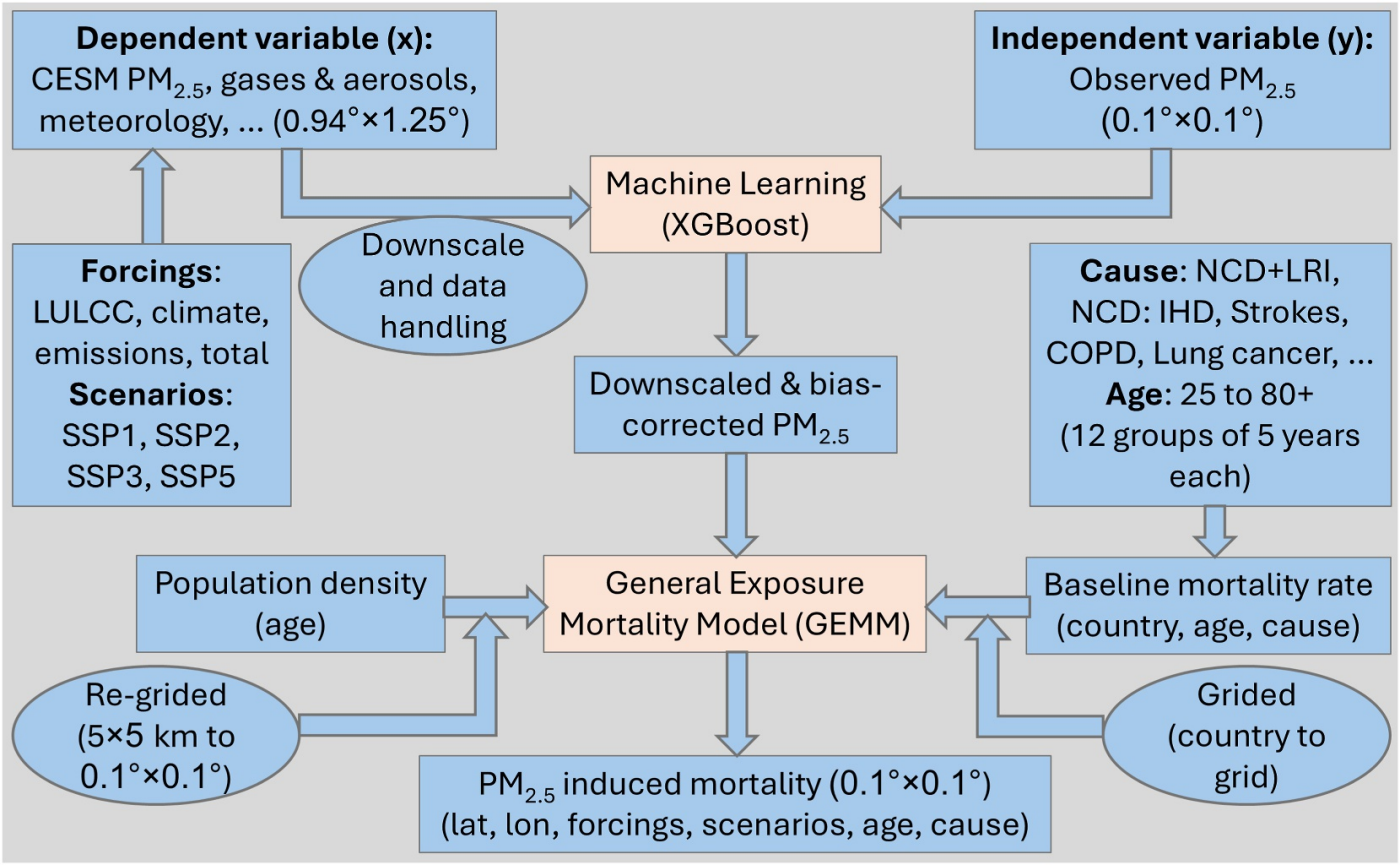
Schematic framework of the integrated modeling approach.

### 2.2 Community Earth System Model (CESM)

We obtained simulated PM_2.5_ using the CESM version 2.1.3 (Bhattarai et al., 2024b; Danabasoglu et al., 2020). CESM is a coupled Earth system model that simulates interactions among the different Earth components, including atmosphere, land, ocean, and ice. The atmospheric component uses the Community Atmosphere Model version 4 chemistry (CAM4‐chem), based on the Model for Ozone and Related chemical Tracers, version 4 (MOZART‐4) chemical mechanism (Emmons et al., 2010). Meteorology is dynamically simulated, while anthropogenic and biomass burning emissions and greenhouse gases are prescribed. Biogenic emissions are calculated online using the Model of Emissions of Gases and Aerosols from Nature (MEGAN).

CESM was run for 17 scenarios (Table S1 in Supporting Information S1): one baseline scenario for 2010 and 16 future scenarios for 2050 (Bhattarai et al., 2024a). Each simulation was conducted for 15 model years, with the first five years considered spin‐up and the subsequent 10 years averaged to minimize interannual variability. The present study employs a time‐slice experimental design, in which the 2010 and 2050 simulations represent equilibrium climate and emissions conditions rather than a transient evolution between the two periods. Accordingly, the 2010 simulation serves as a common reference state for all factorial experiments, enabling the independent contributions of future LULCC, climate change, and emissions to PM_2.5_ concentrations and associated health impacts to be quantified consistently. The 16 future cases represent simulations with three individual forcings and their combined effects (land use and land cover change—LULCC, climate, emissions, and total) for each of four SSPs. All SSPs refer to SSP–RCP combinations consistent with CMIP6 (SSP1‐2.6, SSP2‐4.5, SSP3‐7.0, SSP5‐8.5), hereafter referred to as SSP1, SSP2, SSP3, and SSP5. The three drivers (LULCC, climate, emissions) are isolated by holding the other two drivers at 2010 levels while prescribing the 2050 condition for the driver of interest. For example, the LULCC‐only simulation uses 2050 land use with 2010 climate and 2010 emissions; the climate‐only simulation uses 2050 climate with 2010 land use and 2010 emissions; and the emissions‐only simulation uses 2050 emissions with 2010 LULCC and 2010 climate. The combined (total) simulation prescribes all three drivers at 2050 conditions. The difference between each future simulation and the 2010 baseline isolates the effect of that driver.

The three forcing factors influence PM_2.5_ through distinct physical and chemical mechanisms. LULCC modifies land surface properties (e.g., albedo, surface roughness, and leaf area index), thereby affecting dust emissions, BVOC emissions, and dry deposition velocities. Climate change alters temperature‐dependent gas‐particle partitioning, photochemical reaction rates, precipitation scavenging, atmospheric stagnation, and natural emissions (e.g., BVOC and mineral dust). Anthropogenic and biomass burning emissions are prescribed according to SSP scenarios. Detailed model settings, experimental design, and factorial forcing impact on PM_2.5_ are described previously (Bhattarai et al., 2024b).

### 2.3 Satellite‐Derived PM_2.5_ Observations

The target data set for ML training and validation is the high‐resolution (0.1° × 0.1°) global surface PM_2.5_ concentration estimate from Van Donkelaar et al. (2021). This data set was derived by combining aerosol optical depth (AOD) retrievals from multiple satellite instruments (MODIS, SeaWiFS, MISR) with the GEOS‐Chem chemical transport model and ground‐based observations, using a geographically weighted regression. It provides monthly averaged PM_2.5_ concentrations from 1998 to 2021 and is widely used as a benchmark for model evaluation and exposure assessment.

### 2.4 XGBoost Machine Learning Model

ML models are increasingly employed to reduce biases in model‐simulated results, particularly in atmospheric chemistry (e.g., Mao et al., 2024). Biases in chemistry‐climate models such as CESM often arise from incomplete physical representations, uncertainties in input parameters (e.g., emissions inventories), parameterization schemes, and coding errors (Brasseur & Jacob, 2017). ML approaches, including the XGBoost algorithm (Chen & Guestrin, 2016), offer a powerful means to minimize these biases by leveraging high‐resolution observational data within a data‐driven framework. XGBoost is a highly efficient gradient‐boosting algorithm that builds an ensemble of decision trees additively, optimizing performance while preventing overfitting through regularization. Its strengths include handling large, high‐dimensional data sets, modeling complex non‐linear relationships, and strong generalization performance.

We trained the XGBoost model to statistically bias‐correct and spatially refine CESM‐simulated PM_2.5_ concentrations using the 0.1° satellite‐derived PM_2.5_ product of Van Donkelaar et al. (2021) as the reference. The model establishes nonlinear statistical relationships between coarse‐resolution CESM atmospheric variables and the fine‐resolution PM_2.5_ observations to generate statistically bias‐corrected and spatially refined PM_2.5_ fields. The approach is intended as a statistical bias‐correction framework rather than a physically based downscaling method because it does not explicitly resolve sub‐grid processes or incorporate independent high‐resolution predictors such as land‐use characteristics or emission inventories. Predictor variables included simulated PM_2.5_, major aerosol species (BC, OC, NH_4_NO_3_, SO_4_, SOA, sea salt, and dust), gas‐phase species (NO, OH, HO_2_, RO_2_, and isoprene), AOD, meteorological variables (surface temperature, pressure, planetary boundary layer height, latent and sensible heat fluxes, wind speed, relative humidity, precipitation, and surface albedo), together with latitude and longitude. A complete list of predictor variables, units, and definitions is provided in Table S2 in Supporting Information S1. CESM outputs were bilinearly interpolated to a 0.1° × 0.1° resolution to match the targeted satellite data grid. The monthly baseline data set was randomly split into 80% training and 20% testing data sets. Hyperparameters were optimized using Optuna with 5‐fold cross‐validation performed exclusively within the training data set. The final model was then retrained using the optimized hyperparameters on the complete training data set and evaluated only on the held‐out test data set. Details of the bias correction and ML performance are discussed in Section 3.1. This approach assumes that present‐day bias structures remain approximately stationary under future climate and emission regimes, an assumption widely adopted in scenario‐based air quality studies but still subject to uncertainty.

Because the monthly samples were randomly partitioned into training (80%) and test (20%) sets rather than blocked by location or time, this split represents a random, spatially and temporally non‐independent validation rather than an independent test of spatial or temporal transferability. Grid cells assigned to the test set often share locations (or nearby locations) and time periods with training cells, so the reported test metrics are likely more favorable than those expected for fully out‐of‐sample locations or times. An independent evaluation of spatial or temporal extrapolation would require geographically or temporally blocked cross‐validation, which is not performed here; the test statistics in Section 3.1 should therefore be interpreted accordingly.

### 2.5 Health Impact Assessment

We used the GEMM to estimate the number of premature deaths attributable to long‐term exposure to PM_2.5_ (Burnett et al., 2018). We calculated age‐specific PM_2.5_‐attributable mortality from noncommunicable diseases and LRI (NCD + LRI) for the adult population (>25 years) using the hazard ratio function (Equation 1):

(1) $$\text{HR}=\exp\left\{\frac{\theta \;\ln\left(1+\frac{z}{\alpha }\right)}{1+\exp\left(\frac{µ-z}{\nu }\right)}\right\}$$

where *θ* is age‐cause‐specific exposure‐response coefficient, and *α*, *μ*, and *ν* are cause‐specific empirical parameters obtained from Burnett et al. (2018). Parameter *z* is the difference in PM_2.5_ concentration above a minimum threshold of 2.4 μg m^−3^, below which no health effect is assumed (*z* = max (0, PM_2.5_ − 2.4 μg m^−3^)).

Gridded mortality (*M*, deaths per grid cell) was estimated using Equation 2.

(2) $$M=\frac{\text{HR}-1}{\text{HR}}\times B\times P\times A$$

where *B*, *P*, and *A* are the baseline mortality rate (deaths per 100,000 persons), exposed adult population density (persons/km^2^), and grid cell area (km^2^), respectively. Mortality estimates (*M*) represent the absolute number of attributable deaths per grid cell. For the primary analysis, baseline mortality rates and age‐specific population were fixed at their 2010 values to isolate the effects of changes in PM_2.5_ concentrations. A complementary analysis using SSP‐specific projected population is presented in Section 3.7 to evaluate the influence of future demographic change.

The change in mortality (Δ*M*) attributable to changes in PM_2.5_ between 2010 and 2050 for each forcing and SSP was calculated using Equation 3.

(3) $$\Delta M=M_{2050}-M_{2010}$$

To isolate the effects of changing PM_2.5_ concentrations, we held baseline mortality rates (*B*) and population density (*P*) constant at their 2010 levels throughout the analysis. This approach clarifies the health impact of pollution changes alone, separate from demographic or epidemiological shifts. Baseline mortality rates, obtained from the GBD (https://vizhub.healthdata.org/gbd‐results/), are country‐specific values for 12 age groups (25–29 to 80+ years) and six causes (NCD, COPD, IHD, stroke, lung cancer, LRI). These rates are uniformly applied to all grid cells within a given country. Gridded population data for 2010 were obtained from the 5 km × 5 km Gridded Population of the World version 4 (GPWv4, https://sedac.ciesin.columbia.edu/data/collection/gpw‐v4), which provides age‐specific population counts consistent with national censuses data (Center for International Earth Science Information Network ‐ CIESIN ‐ Columbia University, 2018). To ensure conservation of population during regridding, population density was first converted to absolute population counts using the source grid‐cell area, remapped to the 0.1° × 0.1° resolution using area‐weighted conservative interpolation, then converted back to population density by normalizing with the target grid‐cell area. This procedure preserves total population to within 0.01%.

To quantify uncertainty in these estimates, we performed a Monte Carlo simulation with 10,000 iterations. In each iteration, we resampled only the two inputs for which published uncertainty estimates were available: the GEMM exposure‐response coefficient *θ* and the baseline cause‐specific mortality rate *B*. For each of the 72 cause‐age combinations (6 causes × 12 age groups), *θ* was drawn from a normal distribution centered on its published value with the standard error reported by Burnett et al. (2018), and a single draw was applied to all grid cells belonging to that cause and age group. Baseline mortality rates were drawn independently for each country, age group and cause from a lognormal distribution. Because the GBD “val” field represents the arithmetic mean of *B*, we parameterized the lognormal on that mean: its log‐scale standard deviation was set to *σ* = (ln (*B*_upper_)−ln (*B*_lower_))/(2 × 1.96), which reproduces the published 95% bound, and its log‐scale location was set to ln (*B*_val_)−*σ* ^2^/2, so that the mean of the simulated distribution equals *B*_val_. The remaining GEMM parameters (*α*, *μ*, *ν*) were held fixed, as no uncertainty estimates were available for them. No fractional uncertainty was derived or applied; the interval width reflects the disease‐specific standard error of *θ* and the disease‐specific GBD bounds, and therefore differs across causes. All draws were generated independently, with the exception that each cause‐age *θ* draw was shared across all grid cells to prevent spatial averaging from artificially narrowing the interval. We report the 2.5th and 97.5th percentiles of the simulated distribution as 95% uncertainty intervals, because they represent the spread arising from uncertainty in these input parameters rather than sampling error of an estimator. The central estimates were computed separately using *θ* and *B* at their point estimates, so the simulation supplies only the interval. These intervals propagate uncertainty in *θ* and *B* alone; they exclude uncertainty in the PM_2.5_ exposure field (including uncertainty introduced by the ML bias correction), uncertainty in CESM internal variability or inter‐model spread, uncertainty in the SSP pathway assumptions, or uncertainty in the population projections.

### 2.6 Future Demographic Analysis

The primary analysis isolates the effects of future PM_2.5_ changes by holding population age structure and baseline mortality rates fixed at 2010 values. To evaluate the influence of demographic change, we additionally performed a parallel analysis using SSP‐specific projected total population (Wang et al., 2024) while preserving the 2010 gridded age distribution because globally gridded age‐specific SSP projections are currently unavailable.

## 3 Results

### 3.1 Bias Correction and Model Performance

Accurate representation of surface PM_2.5_ concentrations is essential for reliable estimation of population exposure and attributable mortality. Figure 2 compares raw CESM‐simulated, satellite‐derived observational, and ML bias‐corrected PM_2.5_ fields for the 2010 baseline. The native CESM output exhibits pronounced and spatially systematic biases, overestimating PM_2.5_ across arid and semi‐arid regions (e.g., Sahara, Middle East, Australia) while underestimating concentrations in tropical forested regions (e.g., South America and Central Africa) and densely populated pollution hotspots in South and East Asia.

**Figure 2:**
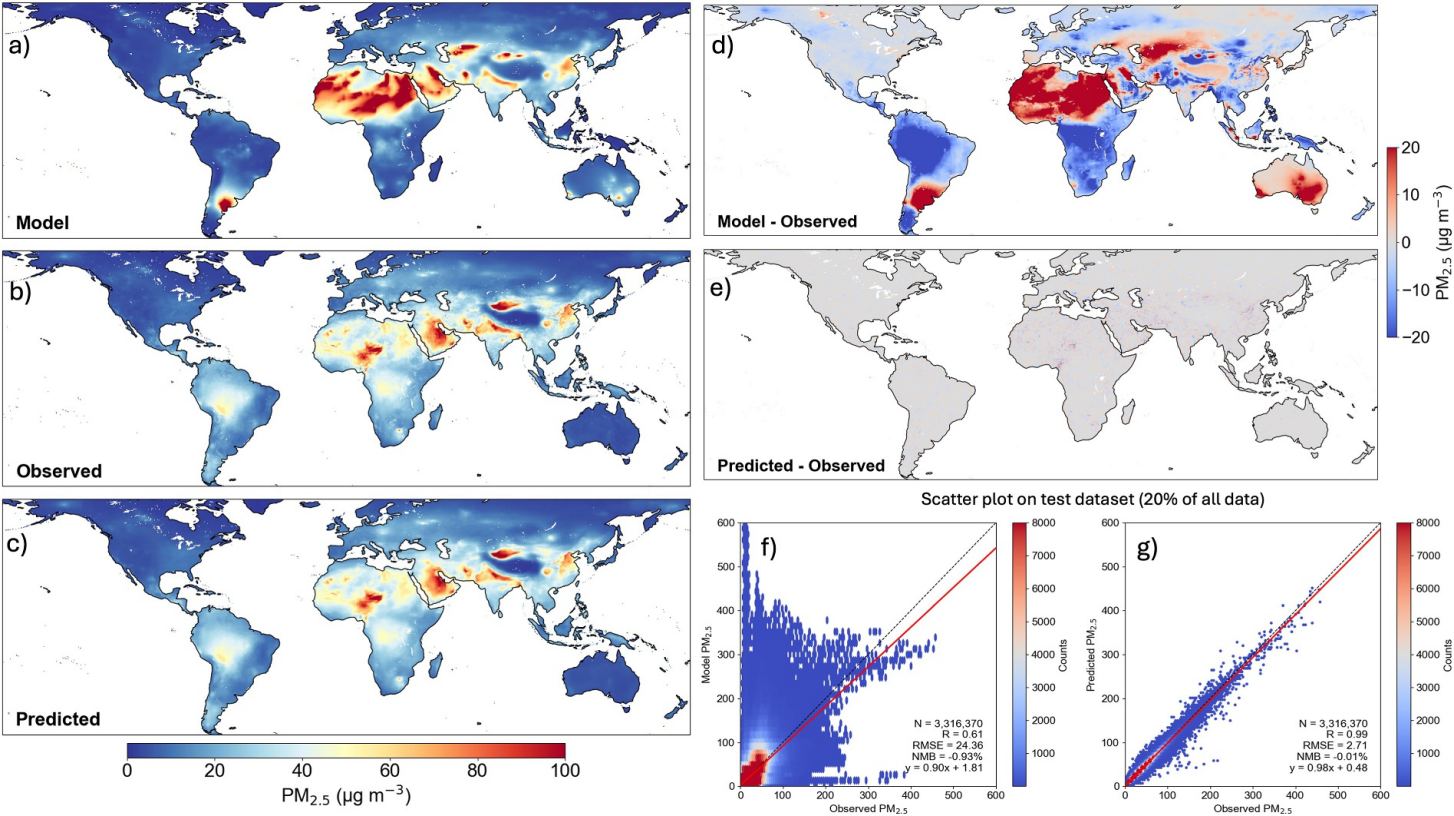
Spatial variations of (a) modeled, (b) observed, and (c) predicted (ML‐bias corrected) PM_2.5_ in the 2010 baseline scenario. Panels (d) and (e) show the difference in model and predicted PM_2.5_ against observation. Density scatter plot of observation against (f) model and (g) predicted is shown with statistical analysis information. Dashed and solid lines represent the 1:1 (*x* = *y*) and regression, respectively.

These biases are consistent with known limitations of global aerosol models, including excessive dust emissions in arid environments and incomplete representation of secondary organic aerosol formation and biomass burning in tropical and highly polluted regions (Bhattarai et al., 2024b; Jo et al., 2023; Kok et al., 2021; Li et al., 2022). Left uncorrected, such biases would disproportionately distort exposure estimates in regions with large populations and high baseline disease burdens, directly propagating into mortality estimates.

Application of XGBoost‐based bias correction substantially improves model fidelity. The spatial correlation (*R*) with observations increases from 0.61 to 0.99 (*R* ^2^ ≈ 0.98), while RMSE decreases from 24.36 to 2.71 μg m^−3^ on an independent test data set comprising 20% of samples that were excluded from all stages of model training and hyperparameter optimization. Importantly, the corrected fields reveal sharp spatial gradients and urban‐regional contrasts that are largely absent in the coarse‐resolution CESM output, particularly over the Indo‐Gangetic Plain, eastern China, and other major population centers. These improvements are critical for health impact assessment, as mortality is driven primarily by exposure extremes in densely populated regions rather than by regional mean concentrations.

Feature importance analysis (Figure S1 in Supporting Information S1) indicates that the ML model primarily adjusts magnitude biases while preserving physically consistent spatial patterns. Simulated PM_2.5_ remains the dominant predictor (33.5%), supplemented by geographic coordinates (latitude 11.8%, longitude 7.9%), aerosol components (black carbon: BC 8.2%; dust 4.8%), and meteorological variables such as wind speed (3.0%). Taken together, these results show that the ML framework substantially reduces the large‐magnitude systematic biases of the raw CESM fields and bridges the scale gap between Earth system models and exposure assessment, providing a high‐resolution (0.1°) PM_2.5_ data set for subsequent mortality analysis.

### 3.2 Global Burden of PM_2.5_‐Attributable Mortality

Applying the GEMM to bias‐corrected 2010 PM_2.5_ fields reveals a substantial global mortality burden of approximately 6.13 million premature deaths attributable to long‐term PM_2.5_ exposure from NCD + LRI (95% UI: 5.77–6.48 million; Table S4 in Supporting Information S1). This estimate exceeds the combined mortality from five specific causes (5‐COD: IHD, stroke, COPD, lung cancer, LRI; 4.71 million) by 30%, reflecting the broader disease coverage of the NCD + LRI category. Our estimate is approximately 31% lower than the 8.9 (range 7.5–10.3) million deaths reported by Burnett et al. (2018) for 2015 using GEMM. This difference likely reflects a combination of factors, including in differences in the baseline period (2010 vs. 2015), PM_2.5_ exposure fields, population data sets and processing, and methodological differences in the health impact assessment. Our estimated mortality is, however, slightly higher than the IER‐based estimate (Cohen et al., 2017) and closer to that reported by Murray et al. (2020). All reported future changes in mortality reflect changes in PM_2.5_ exposure only, with population and baseline mortality rates held constant at 2010 levels to isolate pollution‐driven effects.

The global burden is highly unevenly distributed. Mortality is concentrated in regions where elevated PM_2.5_ coincides with high population density and baseline mortality rates (Figure S2 and S3a, S3b in Supporting Information S1), creating a “double burden” most evident in South and East Asia (Figure 3a). Cardiovascular diseases dominate cause‐specific mortality, with IHD and stroke collectively accounting for over half of the attributable deaths (Figure 3b), underscoring the central role of cardiopulmonary pathway in PM_2.5_ toxicity.

**Figure 3:**
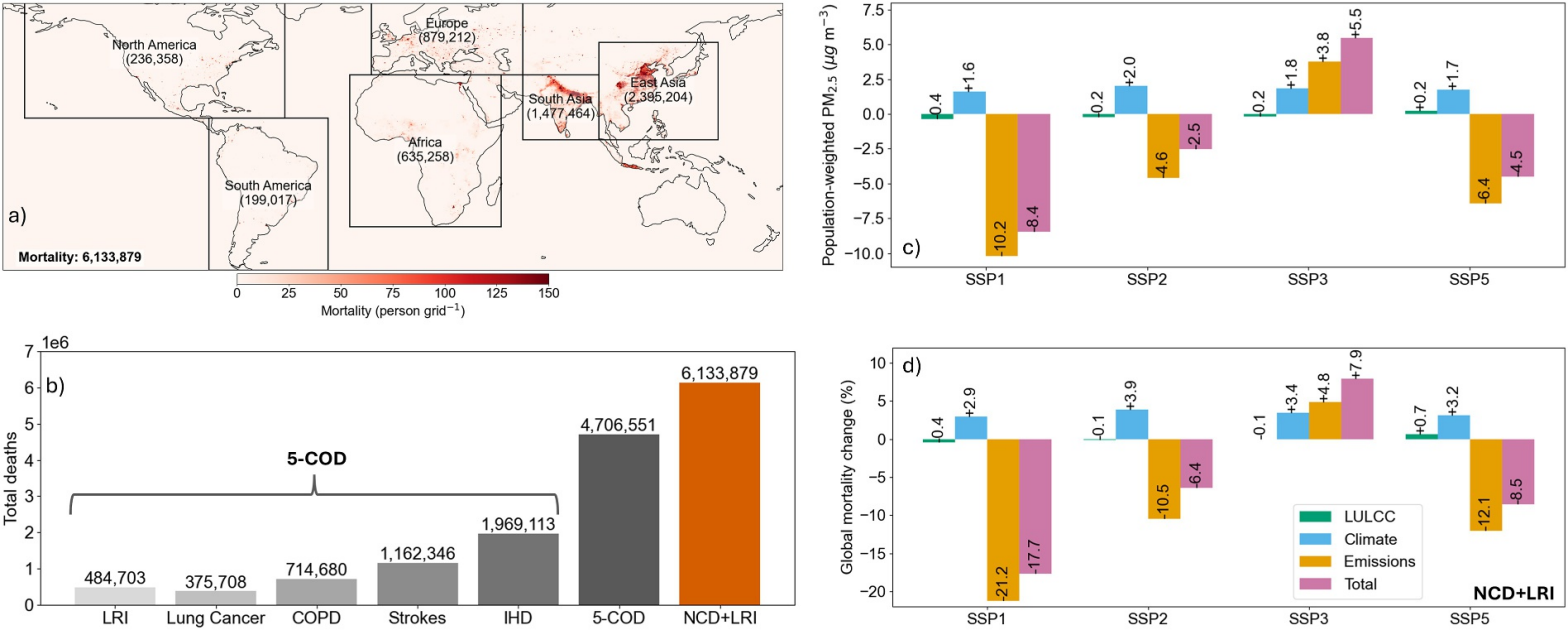
Baseline (2010) information of (a) Spatial variation of NCD + lower respiratory infections (LRI) attributed PM_2.5_ induced mortality and (b) cause‐specific mortality for different diseases. Future changes in (c) population‐weighted PM_2.5_ and (d) NCD + LRI induced global mortality are shown for various socioeconomic pathways and forcing factors. All mortality refers to NCD + LRI unless otherwise specified. Rectangular boxes in panel (a) indicate different regions, and the numbers inside each box indicate total mortality in the region.

Future changes in population‐weighted global mean PM_2.5_ are strongly correlated with changes in global mortality across SSPs and forcing factors (Figures 3c and 3d). LULCC exerts only a minor influence at the global scale, whereas climate change consistently increases PM_2.5_‐related mortality across all SSPs. Anthropogenic and biomass burning emissions remain the dominant driver of future outcomes. SSP1 yields the largest global mortality reduction, SSP3 is the only pathway associated with a net increase in global mortality, and SSP2 and SSP5 show intermediate responses. Notably, climate‐driven increases in PM_2.5_ partially offset the benefits of emission reductions across all pathways, quantifying a persistent “climate penalty” on air quality and health.

### 3.3 Spatial Patterns of PM_2.5_ Exposure and Mortality

Changes in PM_2.5_ concentrations (Figure 4a) and associated mortality (Figure 4b) exhibit pronounced spatial heterogeneity across SSPs and forcing factors. Under LULCC forcing alone, PM_2.5_ changes are modest globally (<±1%), producing relatively small mortality responses. Climate forcing, however, systematically increases PM_2.5_ concentrations and mortality (∼180,000 to 240,000 deaths globally) in many regions, particularly in South and East Asia, driven by altered meteorology, enhanced stagnation, and increased secondary aerosol formation (Bhattarai et al., 2024b). In addition, the dust emission scheme in CESM2 is prognostic and depends on climate (e.g., soil moisture, wind speed), which can drive dust fluxes.

**Figure 4:**
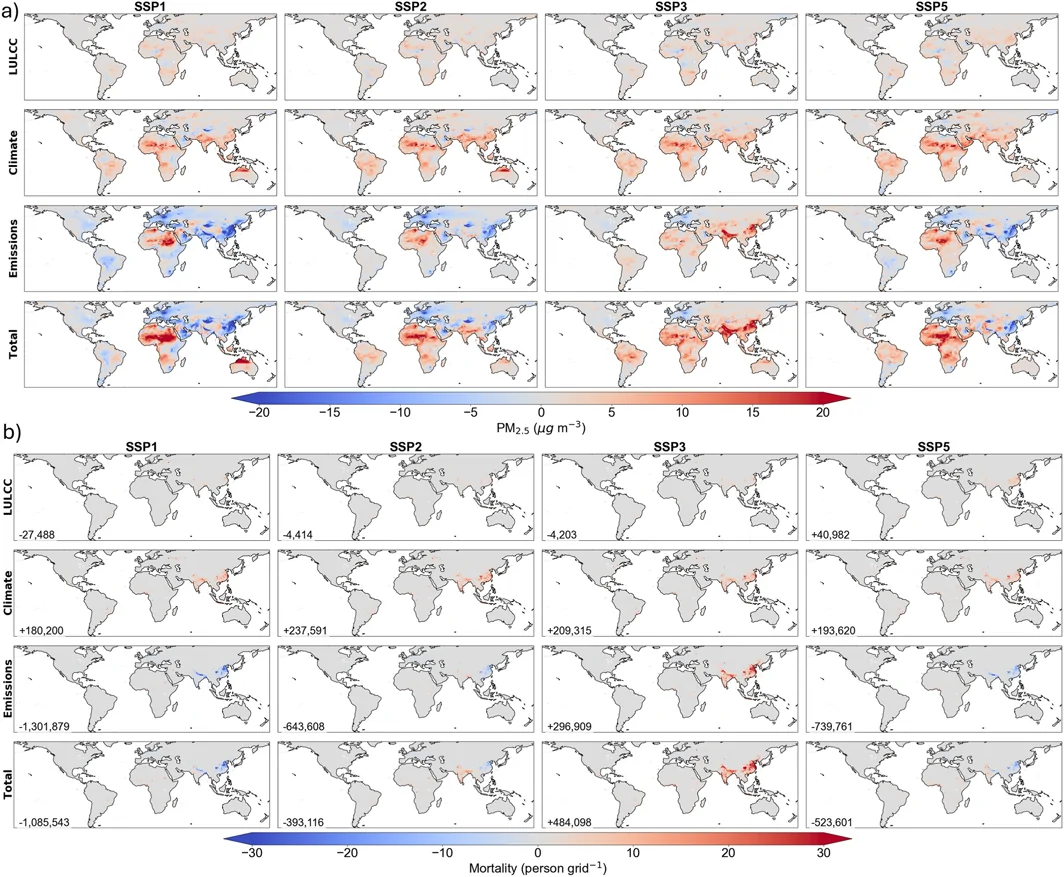
Differences (2050–2010) in (a) PM_2.5_ concentration and (b) PM_2.5_‐induced mortality due to NCD + LRI under individual and combined effects of changes in land use, climate, and emissions for different shared socioeconomic pathway scenarios (SSP1, SSP2, SSP3, and SSP5).

Emissions forcing dominates spatial mortality patterns. Under SSP1, widespread PM_2.5_ reductions across East Asia, Europe, and North America translate into substantial mortality declines (1.3 million). In contrast, SSP3 produces sharp PM_2.5_ increases and mortality escalation (0.30 million) across South and East Asia and parts of Africa, reflecting weak emission controls. SSP2 and SSP5 yield mixed outcomes, with improvements in many industrialized regions but persistent or worsening conditions in developing regions.

The combined (total) forcing reflects the interactions of these drivers and reveals important nonlinearities. The combined effect of LULCC, climate, and emissions was largely additive across SSP scenarios (Table S3 in Supporting Information S1), with deviations between the expected linear sum and the fully coupled simulations ranging from −3.5% (SSP5) to 5.9% (SSP1), suggesting that non‐linear interactions among the three drivers are relatively minor in this system. In several regions, climate‐driven PM_2.5_ increases partially or fully offset emission‐driven improvements, particularly in South Asia under SSP5. These results demonstrate that emission controls alone may be insufficient to deliver sustained health benefits in climate‐sensitive regions without concurrent climate mitigation and adaptation strategies.

### 3.4 Age‐Specific Vulnerability to PM_2.5_ Across SSPs

Age‐stratified mortality results reveal an overwhelming concentration of PM_2.5_‐related health impacts among older populations (≥60 years) (Figure 5 and Figure S4‐S8 in Supporting Information S1). Across all SSPs and forcing factors, this age group accounts for 71%–82% of total mortality changes, indicating that the health burden of PM_2.5_ disproportionately affects older populations because of their greater baseline disease risk and higher age‐dependent susceptibility represented in the GEMM exposure‐response function.

**Figure 5:**
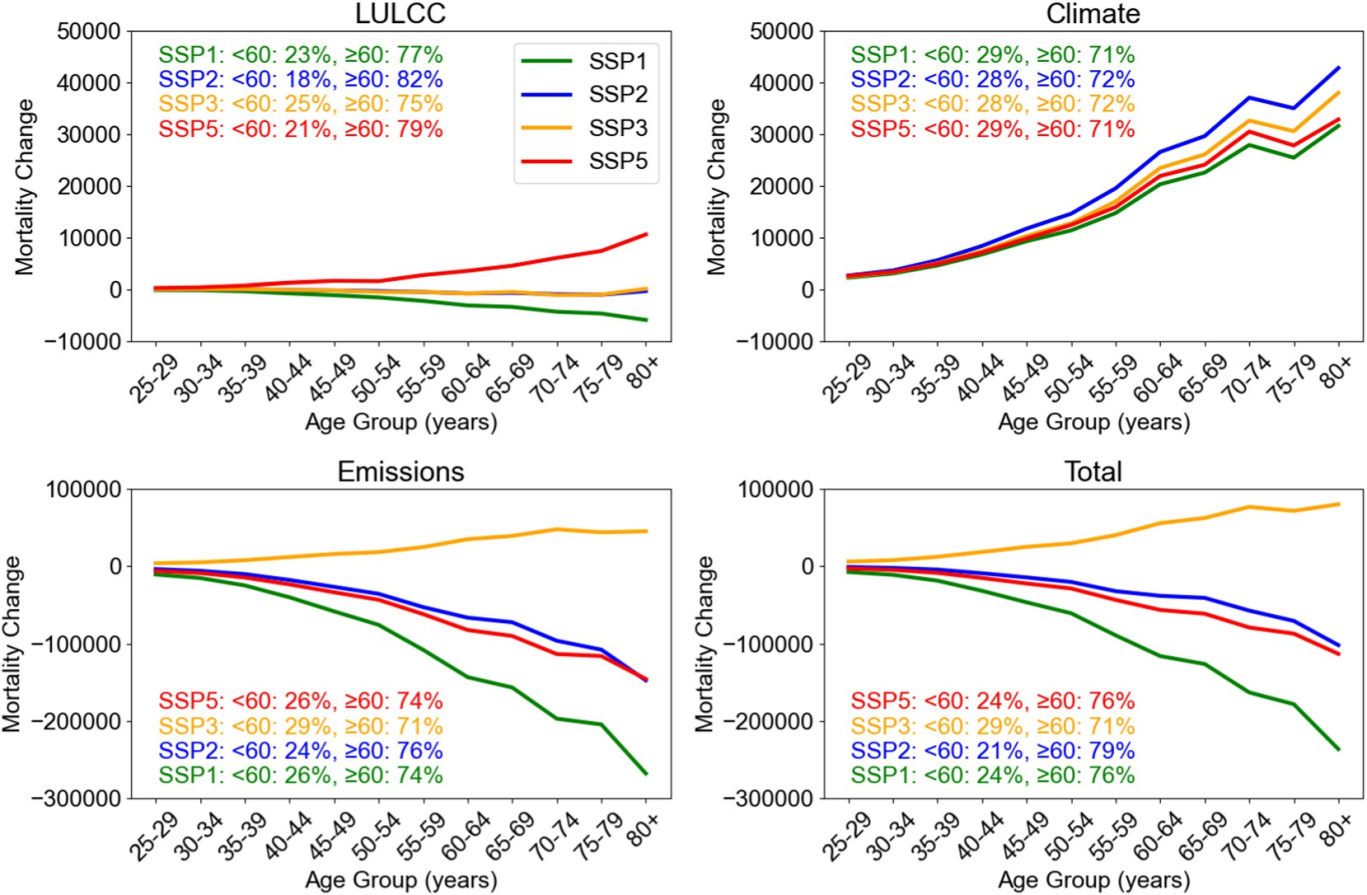
Age‐specific mortality changes due to NCD + LRI for four shared socioeconomic pathways driven by land use (LULCC), climate, emissions, and their combined (total) effects.

Under SSP1, emission reductions produce substantial health benefits, with 74% of avoided deaths occurring among older populations. Conversely, under SSP3, deteriorating air quality leads to increased mortality, of which approximately 74% occurs in the ≥60‐year age group. Mortality changes associated with climate forcing also remain strongly concentrated among older populations, who account for 71%–72% of climate‐related mortality changes despite the smaller overall magnitude, highlighting their heightened physiological vulnerability.

The influence of LULCC on mortality is comparatively modest but exhibits a similar age‐dependent pattern. Overall, these results demonstrate that older populations are consistently the most vulnerable population to PM_2.5_ exposure. Consequently, air quality mitigation must be complemented by public health adaptation strategies—such as improved healthcare access and exposure reduction for older populations—to effectively reduce future mortality.

### 3.5 Regional Attribution of PM_2.5_‐Induced Mortality

#### 3.5.1 NCD + LRI‐Induced Mortality Under Individual Forcings

Regional analysis reveals stark contrast in how different forcing factors shape PM_2.5_‐related mortality (percentage change: Figure 6, absolute change: Figure S9 in Supporting Information S1). Emissions forcing overwhelmingly dominates mortality outcomes in South and East Asia, where population density and baseline disease rates are high. Under SSP1, these regions experience substantial mortality reductions, whereas SSP3 leads to sharp increases, particularly in India and China. East Asia shows the highest mortality increases under SSP3 (+285,480; +11.9%), driven largely by emissions (+8.1%), while the greatest reductions occur under SSP1 (−631, 271; −26.4%). A similar pattern is observed in South Asia, where mortality increases by +193,713 under SSP3, corresponding to a +13.1% increase (emissions only: +10.0%). Europe exhibits consistent reductions in mortality across all SSPs, with the largest decline under SSP1 (−227, 998; −25.9%). North America also experiences mortality reductions under all SSPs, but with a lower magnitude, typically below −17%. This suggests that strict emissions regulations and sustained improvements in air quality are highly effective in reducing PM_2.5_‐induced mortality.

**Figure 6:**
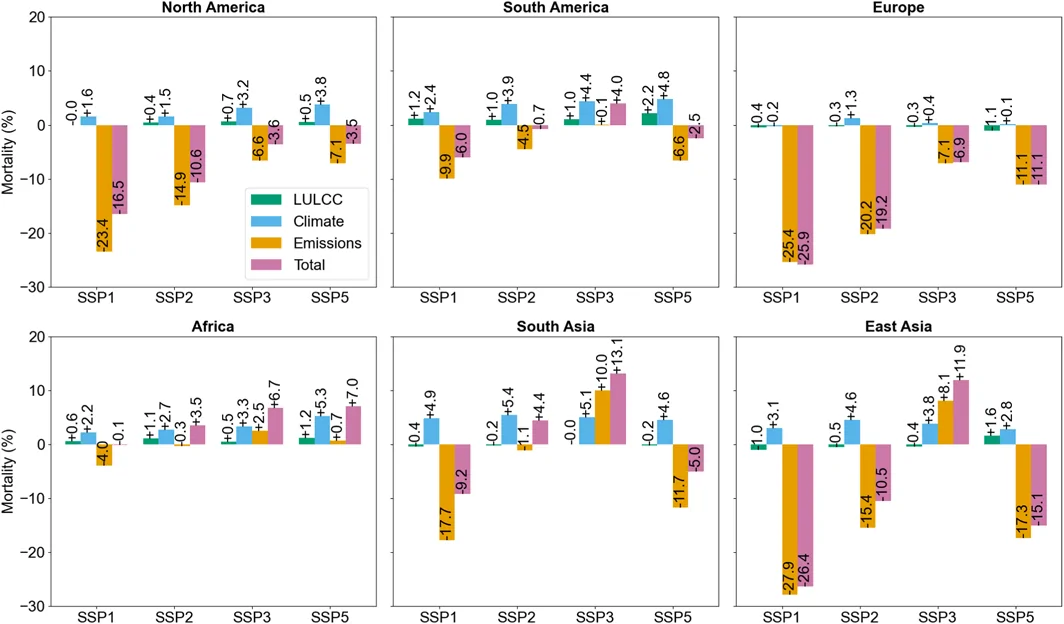
PM_2.5_‐induced mortality difference (%) for various regions under different shared socioeconomic pathway scenarios and different forcing factors.

Climate forcing increases mortality across most regions, with especially strong effects in South and East Asia. In contrast, Europe and North America experience smaller climate‐driven impacts, reflecting lower baseline PM_2.5_ levels and more favorable meteorological conditions. Africa exhibits relatively smaller percentage changes but remains burdened by high baseline mortality, indicating persistent vulnerability. For instance, in Africa under SSP5, mortality increases by +44,745 (+7.0%), largely due to climate effects (+33,509; +5.3%). Similarly, in South America, climate change contributes +2.4%–4.8% increases in all SSPs, most often counteracting the reduced mortality by emissions control.

LULCC‐driven mortality changes are comparatively small globally but regionally heterogeneous. In some areas (e.g., East Asia), land use change modestly reduces mortality under SSP1‐SSP3 but increase it under SSP5, reflecting interactions between land management, dust emissions, and population exposure. The change is noticeable in South America, East Asia, and Africa, where mortality increases up to +2%. In East Asia, land use change alone can result in around +38,000 deaths under SSP5, indicating the substantial role of land cover‐induced air pollution. In North America, Europe, and South Asia, LULCC‐related mortality changes remain relatively lower across all SSPs, reaffirming that emissions and climate are the dominant drivers of air pollution‐related mortality.

#### 3.5.2 5‐COD‐Induced Mortality Under Total Forcings

Analysis of five specific causes of deaths (IHD, stroke, COPD, lung cancer, and LRI) under total forcing reveals that cardiovascular diseases dominate future mortality outcomes across all SSPs (absolute: Figure 7; percentage: Figure S10 in Supporting Information S1). Stroke and IHD show the largest absolute and relative changes, particularly in South and East Asia, consistent with their strong sensitivity to PM_2.5_ exposure. Europe and North America experience widespread reductions across all causes under SSP1 and SSP2, while South Asia shows consistent increases under SSP3. Africa exhibits smaller relative changes but remains burdened by persistent mortality from LRI, highlighting the intersection of air pollution and broader health system challenges.

**Figure 7:**
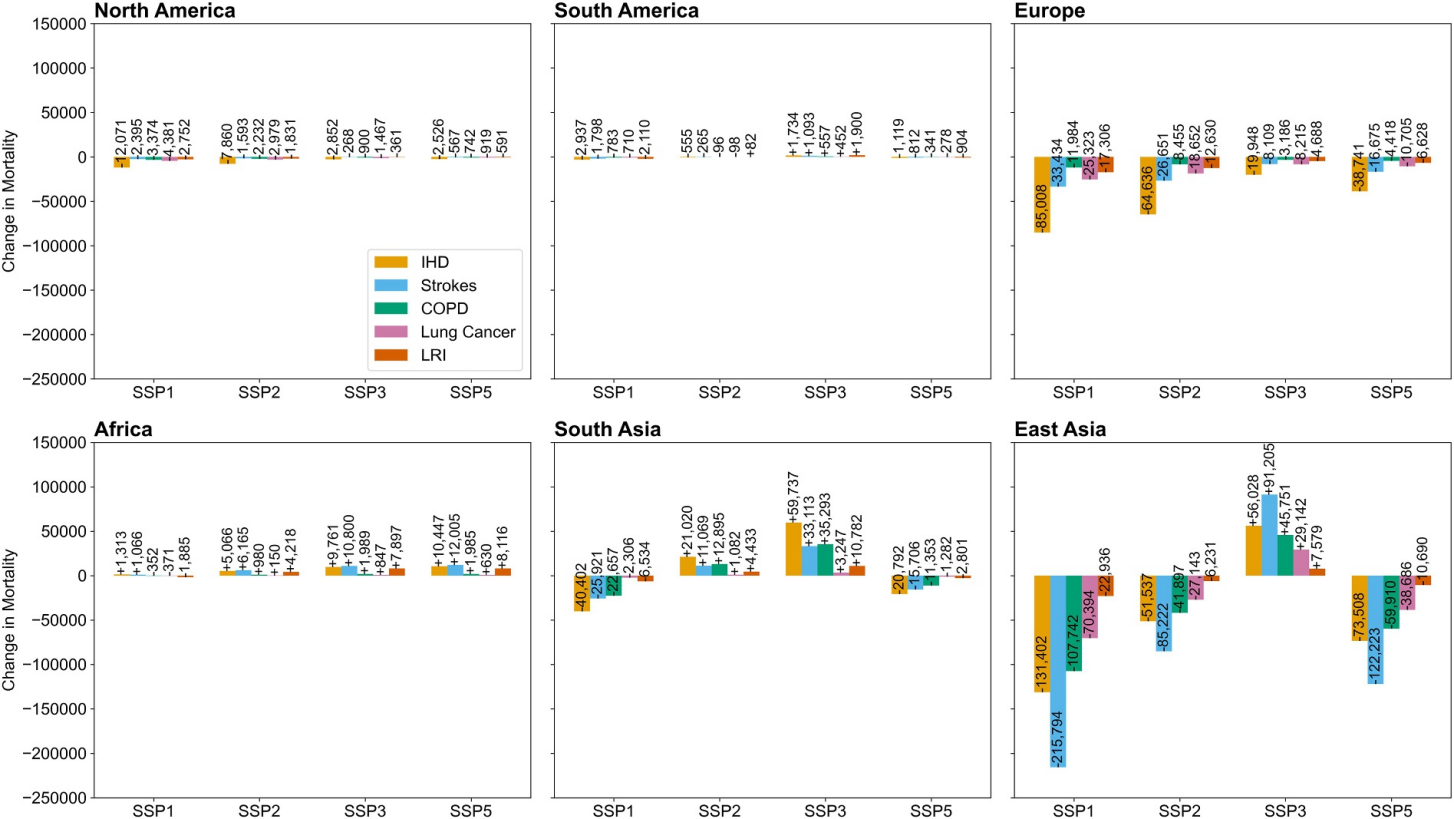
Disease‐specific mortality difference due to total (combined) forcing across different regions (North America, South America, Europe, Africa, South Asia, and East Asia) under Shared Socio‐economic Pathways (SSP1, SSP2, SSP3, SSP5).

While South Asia and Africa exhibit more mixed trends, East Asia experiences the most substantial mortality reductions under SSP1, with strokes, IHD, and COPD declining by around −216,000 (−35%), −131,000 (−23%), and −108,000 (−30%) deaths, respectively. Europe also shows significant mortality reductions, particularly under SSP1, where IHD and strokes contribute to a mortality reduction of around −85,000 (−21%) and −33,000 (−32%), respectively. However, LRI (−38%), lung cancer (−39%), and COPD (−35%) showed the maximum percentage reduction in Europe under SSP1. SSP3 leads to a sharp increase in mortality in East Asia, particularly for strokes (+91,000; 15%) and IHD (+56,000; +10%), whereas in Europe, mortality reductions are still observed but at a lower magnitude compared to SSP1 and SSP2. In North America, mortality consistently declines across all SSPs, but the absolute reductions remain smaller (up to −12,000; −16% deaths due to IHD in SSP1), reflecting a relatively lower baseline burden of PM_2.5_‐induced mortality in this region.

South Asia presents a mixed pattern, with mortality decreasing under SSP1 but increasing significantly under SSP2 and SSP3. Under SSP3, mortality increases exceed around +60,000 (+11%) and +35,000 (+14%) deaths due to IHD and COPD, respectively. However, despite being characterized by fossil‐fuel‐driven economic growth, SSP5 results in mortality reductions of around −50,000 when 5‐COD are combined. This reduction reflects the SSP5 emission inventory prescribed in Earth system models participating in CMIP6, which assumes widespread deployment of advanced air pollution control technologies and stringent end‐of‐pipe emission controls that substantially reduce emissions of PM_2.5_ and its precursor species despite continued fossil fuel use (Bhattarai et al., 2024b; Riahi et al., 2017). Africa and South America, in contrast, show relatively smaller changes. Africa exhibits a moderate reduction in mortality under SSP1 but slight increases under SSP2, SSP3, and SSP5. South America experiences minimal variations, with absolute mortality changes remaining below ±3,000 deaths across all scenarios in any of the individual diseases. This leads to ±10% change in mortality over South America and Africa, predominantly driven by strokes. As such, Europe shows a more balanced and widespread reduction across all diseases, with mortality declining up to 39% (East Asia: up to 35% decline) under SSP1. North America also experiences a substantial percentage decrease of around 11%–24% under SSP1 and SSP2, while in South Asia, mortality increases exceed +8% under SSP3 for all diseases, highlighting its vulnerability to policy inaction. Percentage change in Africa remains small, with fluctuations ranging from −3% to +10%, suggesting a persistent burden of air pollution.

### 3.6 National‐Scale Inequality in PM_2.5_ Mortality

#### 3.6.1 Country‐Level Mortality Burdens

At the national scale, China and India dominate global PM_2.5_‐attributable mortality due to their large populations and persistent air quality challenges (Figure 8). Baseline mortality exceeds 2 million deaths in China and 1.1 million in India, far surpassing other countries. Countries like Russia, Pakistan, USA, Indonesia, and Bangladesh also feature prominently in the baseline, reflecting their vulnerability to current health and pollution issues. Four inset plots in Figure 8 highlight future mortality projections under different SSP scenarios, focusing on the top 15 countries by mortality due to total forcing. Under future scenarios, these two nations (China and India) remain the largest contributors across all SSPs.

**Figure 8:**
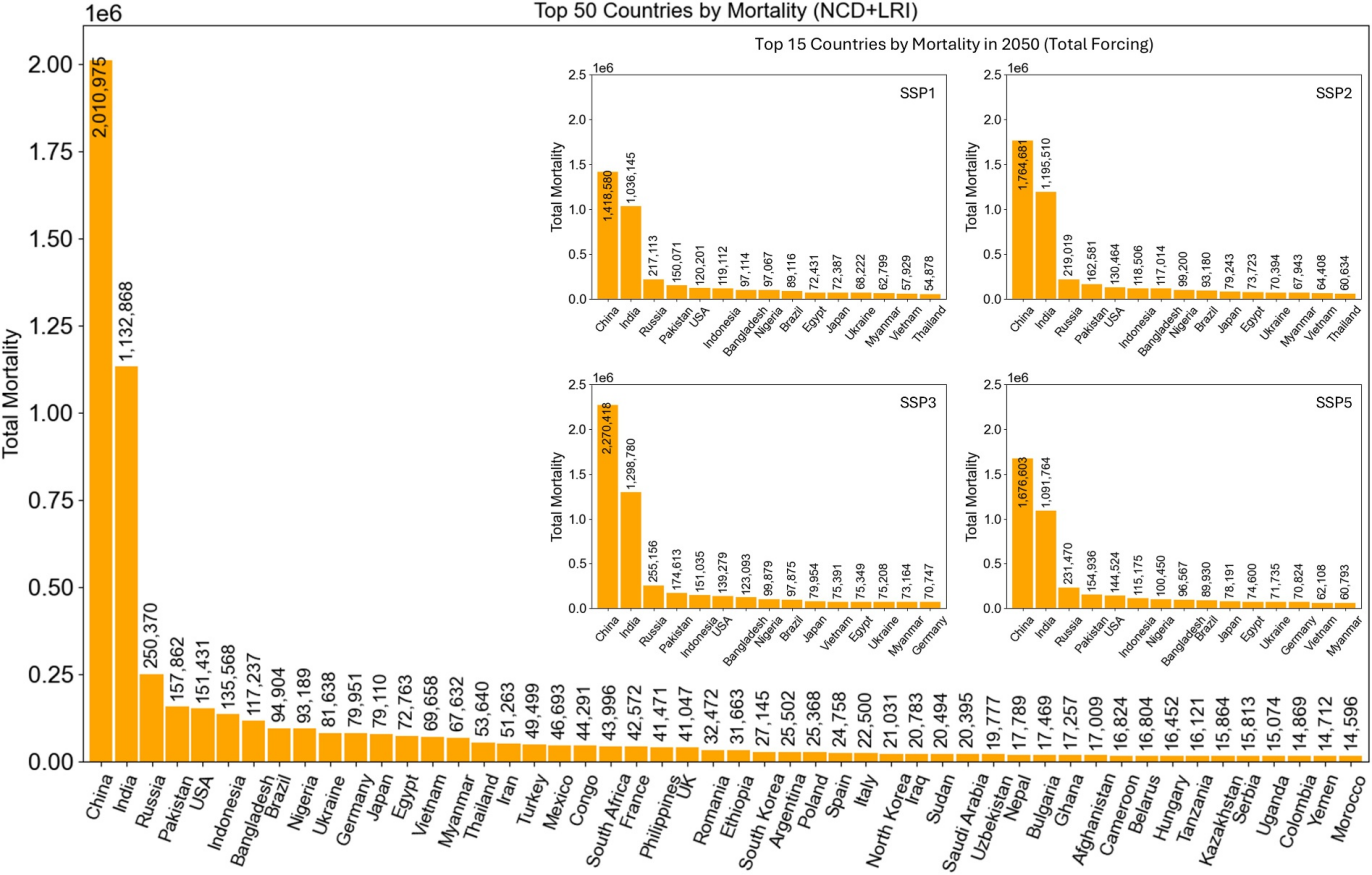
Overview of total mortality attributed to NCD + LRI for the top 50 nations for the baseline. The top 15 nations for future projections due to total forcing under SSP1, SSP2, SSP3, and SSP5 are shown in inset plots.

SSP1 yields the largest mortality reductions globally, particularly in China (reducing from 2.0 million to 1.4 million), reflecting the effectiveness of strong emission controls. SSP3 produces the highest mortality (2.3 million) in China, indicating limited mitigation and worsening air quality. Unlike China, which sees mortality reductions in all SSPs except SSP3, India would experience increased mortality under SSP2 and SSP3. SSP5 shows intermediate outcomes, indicating that technological advances alone cannot fully offset the health impacts of continued fossil fuel reliance.

#### 3.6.2 Mortality Linkage With per Capita Income

PM_2.5_‐induced mortality exhibits a strong inverse relationship with 2010 gross national income (GNI, https://data.worldbank.org/indicator/NY.GNP.PCAP.CD) per capita (Figure 9). Low‐ and lower‐middle‐income countries face substantially higher mortality rates than wealthier nations, both in baseline and future projections. Countries such as India, Pakistan, Bangladesh, Myanmar, Vietnam, Egypt, Congo, and Nigeria consistently exhibit mortality rates exceeding 150 deaths per 100,000 population during baseline, driven by high exposure, rapid industrialization, and limited access to healthcare and pollution control measures. In contrast, high‐income countries, including the USA, UK, France, Italy, Spain, South Korea, and Japan, experience considerably lower mortality rates, typically below 100 per 100,000, reflecting better air quality management, healthcare systems, and economic resilience against environmental stressors.

**Figure 9:**
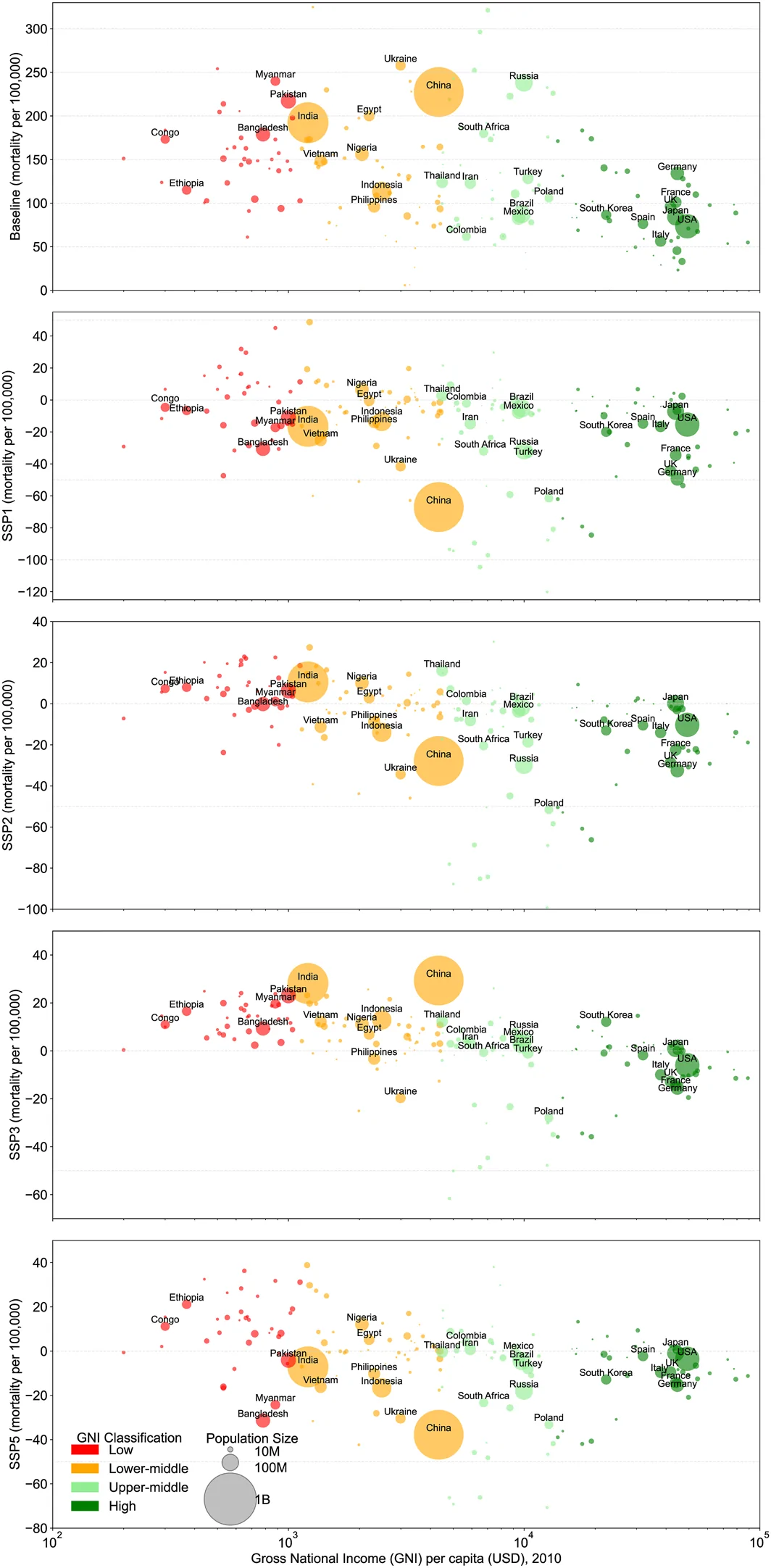
Baseline and future differences in PM_2.5_‐induced mortality under combined forcing (per 100,000) versus gross national income per capita for 2010. Bubble sizes indicate population, and colors represent income classifications (in USD), highlighting disparities in mortality across socioeconomic groups (low: ≤$1,135, lower‐middle: $1,136–4,465, upper‐middle: $4,466–13,845, and high: >$13,845). Only countries with top 30 population size (age 25+) are shown with names.

Under future projections, the extent of mortality reduction or exacerbation largely depends on the socioeconomic pathway. SSP1, which represents a sustainability‐focused trajectory with strong environmental regulations and clean energy transitions, leads to the most pronounced mortality reductions. The benefits are particularly evident in upper‐middle and high‐income countries, such as China and Russia, where aggressive emission controls and technological advancements drive significant improvements. Income (in USD) dependent socioeconomic classification is based on the World Bank data, which classified four groups: low (≤$1,135), lower‐middle ($1,136–4,465), upper‐middle ($4,466–13,845), and high (>$13,845) (https://blogs.worldbank.org/en/opendata/new‐world‐bank‐group‐country‐classifications‐income‐level‐fy24). However, despite overall progress, lower‐income countries continue to experience relatively high mortality burdens, indicating that while global air quality improvements are beneficial, disparities persist due to variations in governance capacity, infrastructure, and healthcare accessibility. A sensitivity analysis using 2023 GNI per capita (Figure S11 in Supporting Information S1) produces nearly identical country groupings and does not alter the observed inverse relationship between national income and PM_2.5_ mortality.

In contrast, SSP2, which follows a middle‐of‐the‐road trajectory with moderate emission reductions, shows only incremental improvements in mortality rates. While countries such as China and Mexico benefit from gradual declines, regions with weaker environmental policies, including South Asia and parts of Africa, remain highly vulnerable. The scenario suggests that without more ambitious interventions, many developing nations will struggle to achieve substantial reductions in PM_2.5_‐induced mortality, reinforcing the need for more proactive policy measures beyond moderate economic development.

SSP3 presents the most concerning outcome, with mortality rates increasing substantially, particularly in low‐income nations. Weak environmental regulations, continued reliance on fossil fuels, and socioeconomic stagnation contribute to worsening air quality and rising health burdens. The effects are most pronounced in densely populated regions such as South Asia, where industrial growth without adequate pollution control measures exacerbates PM_2.5_ exposure. Notably, even some upper‐middle‐income countries, including China and Russia, show limited progress under this scenario, emphasizing that economic growth alone is insufficient to combat air pollution‐related health impacts without complementary policy interventions.

SSP5, which represents a fossil‐fuel‐driven economy with technological advancements, produces mixed results. While high‐income countries continue to benefit from cleaner technologies and advanced pollution control mechanisms, developing nations still experience elevated mortality due to unchecked industrial expansion. The findings suggest that while technological progress can mitigate air pollution impacts in wealthier nations, it does not automatically translate into health benefits for lower‐income countries unless accompanied by strong regulatory frameworks and equitable access to clean energy solutions.

Overall, while economic growth is associated with lower mortality, it is not a standalone solution for air pollution‐related health risks. Countries with strong environmental policies, such as those projected under SSP1, demonstrate the most significant mortality reductions, whereas those under weak governance (SSP3) face worsening outcomes. These findings underscore the importance of integrated mitigation strategies that combine emissions reductions, sustainable land‐use practices, and climate adaptation measures. Targeted policy interventions are essential to bridge the health disparity gap between high‐ and low‐income nations, ensuring that future air quality improvements translate into equitable health benefits across all socioeconomic groups.

### 3.7 Influence of Future Demographic Change

To evaluate the influence of demographic change on future health burdens, we repeated the mortality calculations using SSP‐specific projected population while preserving the 2010 age structure. This analysis isolates the contribution of changes in total population size, while avoiding additional uncertainty associated with unavailable gridded future age distributions.

Figure 10 shows that incorporating SSP‐specific population substantially modifies the magnitude of projected premature mortality compared with the fixed‐demographic analysis. Under the original framework, changes in PM_2.5_ exposure alone alter global mortality by approximately −1.09 million (SSP1), −0.39 million (SSP2), +0.48 million (SSP3), and −0.52 million (SSP5) relative to the 2010 baseline. However, when future population projections are included, demographic contribution exceeds the environmental effect in all scenarios, indicating that future population dynamics represent a dominant source of change in global PM_2.5_‐attributable mortality.

**Figure 10:**
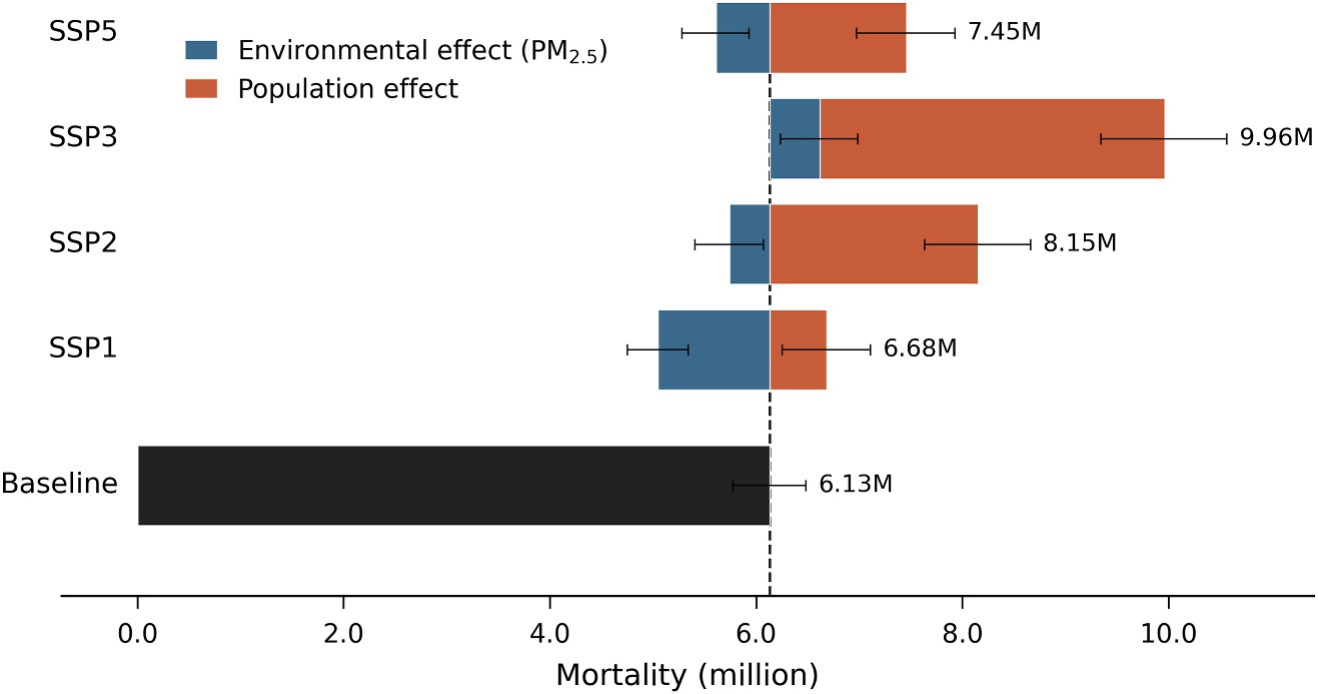
Decomposition of projected NCD + LRI mortality (in millions) under four shared socioeconomic pathway (SSP) scenarios (SSP1, SSP2, SSP3, and SSP5). For each SSP, the total projected mortality is decomposed into the component attributable to changes in ambient PM_2.5_ concentration (blue) and the additional component attributable to SSP‐specific population change (orange). The dashed vertical line indicates the baseline mortality level. Error bars represent 95% uncertainty intervals (UIs) derived from the Monte Carlo analysis.

The combined effects of environmental and population changes produce projected global premature mortalities of 6.68 million (SSP1), 8.15 million (SSP2), 9.96 million (SSP3), and 7.45 million (SSP5), compared with 6.13 million in 2010. Despite the strong influence of demographic change on the absolute mortality burden, the overall scenario ranking remains unchanged. SSP1 continues to yield the lowest mortality because reductions in PM_2.5_ exposure offset much of the increase associated with population growth, whereas SSP3 produces the highest mortality owing to the combined effects of deteriorating air quality and continued population increase. SSP2 and SSP5 exhibit intermediate responses, reflecting different balances between environmental improvements and demographic change.

These findings indicate that future demographic evolution primarily influences the magnitude of PM_2.5_‐attributable mortality, whereas the relative effectiveness of different SSP pathways remains largely controlled by changes in air quality. Consequently, the fixed‐population analysis presented in Sections 3.2–3.6 remains appropriate for isolating the individual contributions of emissions, climate, and land‐use change, while the complementary demographic analysis provides a more realistic estimate of the future health burden.

## 4 Discussion and Conclusions

This study provides a comprehensive, high‐resolution assessment of present‐day and future PM_2.5_‐attributable mortality by integrating Earth system modeling, ML bias correction, and epidemiological analysis within a factorial forcing framework. By explicitly disentangling the roles of emissions, climate change, and land use change across multiple SSPs, we advance the understanding of the mechanisms shaping future air pollution health burdens.

Our results not only confirm that anthropogenic and biomass burning emissions remain the dominant driver of PM_2.5_‐related mortality globally but also reveal that climate change imposes a persistent and non‐negligible penalty on air quality and health. Even under strong emission mitigation pathways, climate‐driven increases in PM_2.5_ offset a meaningful fraction of health gains. This effect is particularly pronounced in South Asia. This finding highlights the need to integrate air quality management with climate mitigation and adaptation strategies rather than treating them as separate policy domains.

The strong concentration of mortality impacts among older populations underscores the importance of demographic dynamics in shaping future health outcomes. As populations age, even modest PM_2.5_ exposures are generally expected to translate into disproportionate mortality burdens. However, our sensitivity analysis preserved the 2010 age structure and varied only total (SSP‐projected) population size, so population aging itself was not explicitly modeled and this exception is therefore an inference rather than a direct result of our analysis. Nevertheless, the sensitivity analysis demonstrates that projected population change can substantially modify mortality burden, indicating that changes in air quality alone do not determine future health outcomes.

Estimates of PM_2.5_‐attributable mortality are sensitive to the choice of exposure data set and modeling approach. Our 2010 baseline estimate (6.13 million) is lower than the 8.9 (range 7.5–10.3) million deaths for 2015 reported by Burnett et al. (2018) using the same GEMM, yet it is slightly higher than the IER‐based estimate of Cohen et al. (2017) and closer to GBD estimates (Murray et al., 2020). Differences among these assessments may reflect multiple factors, including the reference year, the exposure fields and their spatial resolution, population and baseline mortality data sets, and the health‐impact methodology, and we recommend that future work evaluate the effect of spatial resolution jointly with other sources of discrepancy.

The pronounced disparities across regions, countries, and income groups highlight the inequitable distribution of PM_2.5_ health impacts. Sustainable development pathways with strong environmental governance (SSP1) offer the greatest health benefits, whereas pathways characterized by weak regulation and regional rivalry (SSP3) exacerbate both mortality and inequality. Technological advancement alone, as represented by SSP5, is insufficient without robust policy frameworks.

Despite these strengths, several methodological limitations persist. First, the primary analysis used fixed (2010) population age structure and baseline mortality rates to isolate the effects of changes in PM_2.5_ concentrations. Although a complementary analysis using SSP‐specific population projections is presented in Section 3.7, future changes in age structure and baseline mortality rates were not considered because globally consistent gridded data sets are currently unavailable. Consequently, future mortality may be underestimated in regions experiencing rapid population growth and aging (e.g., India and parts of Africa) and overestimated in countries with declining populations (e.g., China and Japan). Second, GEMM excludes children and young adults (<25 years), potentially underestimating the full burden of PM_2.5_ exposure. Third, we treated PM_2.5_ as a bulk pollutant, although its chemical composition and toxicity vary by source and region. Certain components, such as ultrafine particles, metals (e.g., lead, iron, copper), and polycyclic aromatic hydrocarbons have been linked to cardiopulmonary and neurological risks due to their oxidative potential and ability to translocate beyond the respiratory tract (Kelly & Fussell, 2012; Lelieveld et al., 2015; Manisalidis et al., 2020). Fourth, climate change influences wildfires (Bhattarai et al., 2025) and thereby affects PM_2.5_ levels and associated health impacts. This indirect feedback mechanism is not considered here. Fifth, the ML bias‐correction model was evaluated using a random rather than geographically or temporally blocked data split, limiting assessment of its spatial and temporal transferability. The future projections also rely on the assumption that the learned statistical relationships remain approximately stationary under changing climate and emission regimes, introducing additional uncertainty. Finally, baseline mortality rates were applied uniformly across each country because globally consistent gridded mortality data sets are unavailable. This assumption neglects subnational variability in baseline health conditions and may introduce regional biases in gridded mortality estimates, particularly in countries with urban‐rural differences. Future work should incorporate age‐stratified population forecasts, dynamic mortality rates, PM_2.5_ chemical speciation, and climate‐fire‐PM_2.5_ associations, and subnational health statistics to refine estimates of health burden more comprehensively.

In short, this study demonstrates that achieving sustained reductions in PM_2.5_‐related mortality requires simultaneous progress in emission mitigation, climate action, and public health adaptation, particularly in rapidly developing and climate‐sensitive regions. Our factorial analysis indicates that policy priorities differ across regions. South Asia should remain the highest priority for emission reductions because it experiences the largest PM_2.5_‐attributable mortality burden and the strongest climate penalty, indicating that climate adaptation alone cannot offset the benefits of aggressive emission control. East Asia, particularly China, should sustain stringent industrial and energy‐sector emission controls to preserve the substantial health gains projected under SSP1 while minimizing climate‐driven offsets. Africa should prioritize expansion of air‐quality monitoring networks, cleaner household energy, and healthcare capacity, as mortality burdens remain high despite comparatively lower industrial emissions. Europe and North America, where baseline mortality rates are relatively low, should maintain existing emission reductions while addressing the remaining climate‐driven PM_2.5_ burden through integrated climate and air‐quality policies. Finally, our demographic sensitivity analysis demonstrates that future population change can substantially modify projected mortality, highlighting the need to incorporate demographic projections into long‐term air quality management and public health planning. Our high‐resolution data analysis reveals a future of stark contrasts: one path leads toward healthier populations and reduced inequity; the other toward exacerbated suffering and injustice. These differences correspond to millions of lives potentially lost or saved annually. Achieving the healthier future requires moving beyond isolated policies. It demands an integrated grand strategy for planetary health—one that rigorously mitigates emissions, proactively adapts to climate impacts, and relentlessly targets the vulnerabilities of the most at‐risk populations and regions. The integrated framework presented here provides a policy‐relevant tool for identifying priority regions, vulnerable populations, and the most effective intervention strategies under divergent socioeconomic futures. This is not only an environmental or technical imperative but a fundamental moral and public health necessity for the 21st century.

## Conflict of Interest

The authors declare no conflicts of interest relevant to this study.

## Supporting information

Supporting Information S1

## Data Availability

The processed data set underlying the main results of this study is publicly available at Zenodo (Bhattarai et al., 2026). These include the gridded cause‐ and age‐specific PM_2.5_‐attributable mortality estimates used to create the main figures. Due to the large size of the intermediate climate model (CESM) and machine learning data sets, only the processed data sets are archived in the repository; however, intermediate data sets can be made available upon reasonable request.
