# Supplementary material for: High‐Resolution PM2.5 Mortality Projections With Machine Learning Reveal Global Inequities Under Future Climate and Socioeconomic Pathways
Source: Geohealth. 2026 Nov 10;10(11):e2026GH001829. doi: 10.1029/2026GH001829 (PMC13749607; doi:10.1029/2026GH001829)
Supplement: Supplementary file 1 [file GH2-10-e2026GH001829-s001.pdf]

Supporting information

High-resolution $PM_{2.5}$ Mortality Projections with Machine Learning Reveal Global Inequities Under Future Climate and Socioeconomic Pathways

Hemraj Bhattarai[1,2], Maria Val Martin[3,4,5], Xueying Liu[2], Jia Mao[6], Amos P. K. Tai[1,7]

[1] Department of Earth and Environmental Sciences, The Chinese University of Hong Kong, Hong Kong, China

[2] Division of Environment and Sustainability, The Hong Kong University of Science and Technology, Hong Kong, China

[3] Leverhulme Centre for Climate Change Mitigation, School of Biosciences, University of Sheffield, Sheffield, UK

[4] Now at Research Institute of Water and Environmental Engineering (IIAMA), Universidad Politécnica de Valencia, Valencia, Spain

[5] Now at Valencian Foundation for Excellence in Research (ValER), Castelló de la Plana, Spain

[6] Faculty of Applied Sciences, Macao Polytechnic University, Macao, China

[7] State Key Laboratory of Agrobiotechnology and Institute of Environment, Energy and Sustainability, The Chinese University of Hong Kong, Hong Kong, China

Corresponding: amostai@cuhk.edu.hk (Amos P. K. Tai), hemraj@ust.hk (Hemraj Bhattarai)

Contents of this file

Tables S1 to S4

Figures S1 to S11

Table S1. Overview of the 17 CESM factorial simulations used in this study. The baseline simulation represents 2010 conditions, while the 16 future simulations represent individual and combined effects of LULCC, climate, and emissions under four SSPs (SSP1, SSP2, SSP3, and SSP5).

S.N.	Simulation	LULCC	Climate	Emissions	Description
1	Baseline	2010	2010	2010	Present-day control
2	SSP1-L	2050	2010	2010	2050 LULCC only
3	SSP1-C	2010	2050	2010	2050 Climate only
4	SSP1-E	2010	2010	2050	2050 Emissions only
5	SSP1-T	2050	2050	2050	All drivers at 2050
6	SSP2-L	2050	2010	2010	2050 LULCC only
7	SSP2-C	2010	2050	2010	2050 Climate only
8	SSP2-E	2010	2010	2050	2050 Emissions only
9	SSP2-T	2050	2050	2050	All drivers at 2050
10	SSP3-L	2050	2010	2010	2050 LULCC only
11	SSP3-C	2010	2050	2010	2050 Climate only
12	SSP3-E	2010	2010	2050	2050 Emissions only
13	SSP3-T	2050	2050	2050	All drivers at 2050
14	SSP5-L	2050	2010	2010	2050 LULCC only
15	SSP5-C	2010	2050	2010	2050 Climate only
16	SSP5-E	2010	2010	2050	2050 Emissions only
17	SSP5-T	2050	2050	2050	All drivers at 2050

Table S2. Detailed information on variables used for training machine learning model. Some of these variables are not direct model output and are thus calculated offline as indicated below.

Variables	Units	Description (all variables are surface level CESM output)
$PM_{2.5}$	$\mu g\ m^{-3}$	Fine particulate matter of aerodynamic diameter ≤ 2.5 μm
BC	$kg\ kg^{-1}$	Black carbon
OC	$kg\ kg^{-1}$	Organic carbon
NH_4NO_3	$kg\ kg^{-1}$	Ammonium nitrate
SOA	$kg\ kg^{-1}$	Secondary organic aerosols
SO_4	$kg\ kg^{-1}$	Sulfate
Sea Salt	$kg\ kg^{-1}$	Sea salt (SSLT01)
Dust	$kg\ kg^{-1}$	Dust (DST01)
AOD	-	Total Aerosol Optical Depth in visible band
NO	$kg\ m^{-2}\ s^{-1}$	Nitric oxide surface flux
ISOP	$kg\ m^{-2}\ s^{-1}$	Isoprene surface flux
RO_2	$mol\ mol^{-1}$	Peroxy radicals
HO_2	$mol\ mol^{-1}$	Hydroperoxy radicals
OH	$mol\ mol^{-1}$	Hydroxyl radicals
Temperature	K	Surface temperature (radiative)
RH	%	Relative humidity
Pressure	Pa	Surface pressure
Precipitation	$m\ s^{-1}$	Precipitation rate
PBLH	m	Planetary boundary layer height
Wind (10 m)	$m\ s^{-1}$	10 m wind speed

LHFLX	w m^{-2}	latent heat flux
SHFLX	w m^{-2}	sensible heat flux
Albedo	-	Surface albedo: Solar flux at surface [(FSDS-FSNS)/FSDS]
Latitude	°	Grid latitude
Longitude	°	Grid longitude

$PM_{2.5}$ = BC + 1.8OC + 1.375SO_4 + NH_4NO_3 + SOA + Dust + Sea Salt (unit: kg kg^{-1})
BC = CB1_SRF + CB2_SRF
OC = OC1_SRF + OC2_SRF
Precipitation = PRECC + PRECL
Albedo = (FSDS - FSNS) / FSDS

where, we consider the first bin size for dust (DST01) and sea salt (SSLT01). Conversion factor 1.8 converts OC to organic matter and 1.375 converts sulfate to ammonium sulfate. PRECC represents convective precipitation rate and PRECL indicates large-scale precipitation rate. Similarly, FSDS and FSNS represent downwelling solar flux and net solar flux at surface, respectively.

Unit conversion from kg kg^{-1} to μg m^{-3} takes consideration of air density as:
air_MW = 29×10^6 (unit: μg mol^{-1})
R = 8.314 (unit: Pa m^3 mol^{-1} K^{-1}); gas constant
air_density = (air_MW×Pressure) / (R×Temperature) # to change kg/kg to μg m^{-3}
$PM_{2.5}$ (μg m^{-3}) = $PM_{2.5}$ (kg kg^{-1}) × air_density

Table S3. Additive (linear sum: ΔLULCC + ΔClimate + ΔEmission) versus combined effects of land-use and land-cover change (LULCC), climate, and emissions on total cases under four SSP scenarios. Note: All Δ values represent change relative to the present baseline.
Difference = Expected Additive − Observed ΔTotal.
% Difference = (Expected Additive − Observed ΔTotal) / Observed ΔTotal × 100
Negative % difference indicates that the linear sum underestimation the magnitude of change, while positive % difference indicates that the linear overestimates (synergistic interaction).

SSP scenarios	Present	Δ LULCC	Δ Climate	Δ Emission	Expected additive	Observed ΔTotal	Difference (Exp-Obs)	% Difference
SSP1	6133879	-27488	+180200	-1301879	-1149167	-1085543	-63624	+5.86
SSP2	6133879	-4414	+237591	-643608	-410431	-393116	-17315	+4.40
SSP3	6133879	-4203	+209315	+296909	+502021	+484098	+17923	+3.70
SSP5	6133879	+40982	+193620	-739761	-505159	-523601	+18442	-3.52

Table S4. Global premature mortality attributable to ambient $PM_{2.5}$ exposure (millions of deaths per year) for the baseline period and four future scenarios (SSP1, SSP2, SSP3, and SSP5). Values represent the central estimate with 95% uncertainty intervals (UIs) in parentheses, derived from Monte Carlo simulations (10,000 iterations) accounting for uncertainty in GEMM parameters and baseline mortality rates.

	NCD+LRI	IHD	Strokes	COPD	Lung Cancer	LRI
Fixed population to 2010						
Baseline	6.13 (5.77 - 6.48)	1.97 (1.91 - 2.03)	1.16 (0.96 - 1.33)	0.71 (0.56 - 0.84)	0.38 (0.32 - 0.42)	0.48 (0.40 - 0.54)
SSP1	5.05 (4.75 - 5.34)	1.68 (1.63 - 1.74)	0.87 (0.72 - 1.01)	0.56 (0.44 - 0.67)	0.27 (0.23 - 0.30)	0.43 (0.36 - 0.48)
SSP2	5.74 (5.40 - 6.07)	1.86 (1.80 - 1.92)	1.05 (0.87 - 1.21)	0.67 (0.53 - 0.79)	0.33 (0.28 - 0.37)	0.47 (0.39 - 0.52)
SSP3	6.62 (6.23 - 6.99)	2.08 (2.02 - 2.15)	1.30 (1.07 - 1.49)	0.80 (0.63 - 0.93)	0.40 (0.35 - 0.45)	0.51 (0.43 - 0.57)
SSP5	5.61 (5.28 - 5.93)	1.84 (1.78 - 1.90)	1.01 (0.83 - 1.16)	0.64 (0.50 - 0.76)	0.32 (0.28 - 0.36)	0.47 (0.39 - 0.52)
SSP based population to 2050						
SSP1	6.68 (6.25 – 7.10)	2.17 (2.10 – 2.25)	1.10 (0.90 – 1.28)	0.77 (0.59 – 0.93)	0.29 (0.25 – 0.33)	0.69 (0.55 – 0.79)
SSP2	8.15 (7.63 – 8.66)	2.59 (2.51 – 2.68)	1.42 (1.16 – 1.65)	0.99 (0.75 – 1.18)	0.37 (0.32 – 0.41)	0.83 (0.67 – 0.94)
SSP3	9.96 (9.34 – 10.56)	3.12 (3.01 – 3.22)	1.85 (1.51 – 2.13)	1.25 (0.95 – 1.48)	0.46 (0.40 – 0.51)	0.99 (0.79 – 1.11)
SSP5	7.45 (6.97 – 7.92)	2.38 (2.30 – 2.46)	1.26 (1.03 – 1.47)	0.87 (0.66 – 1.04)	0.36 (0.31 – 0.40)	0.76 (0.60 – 0.86)

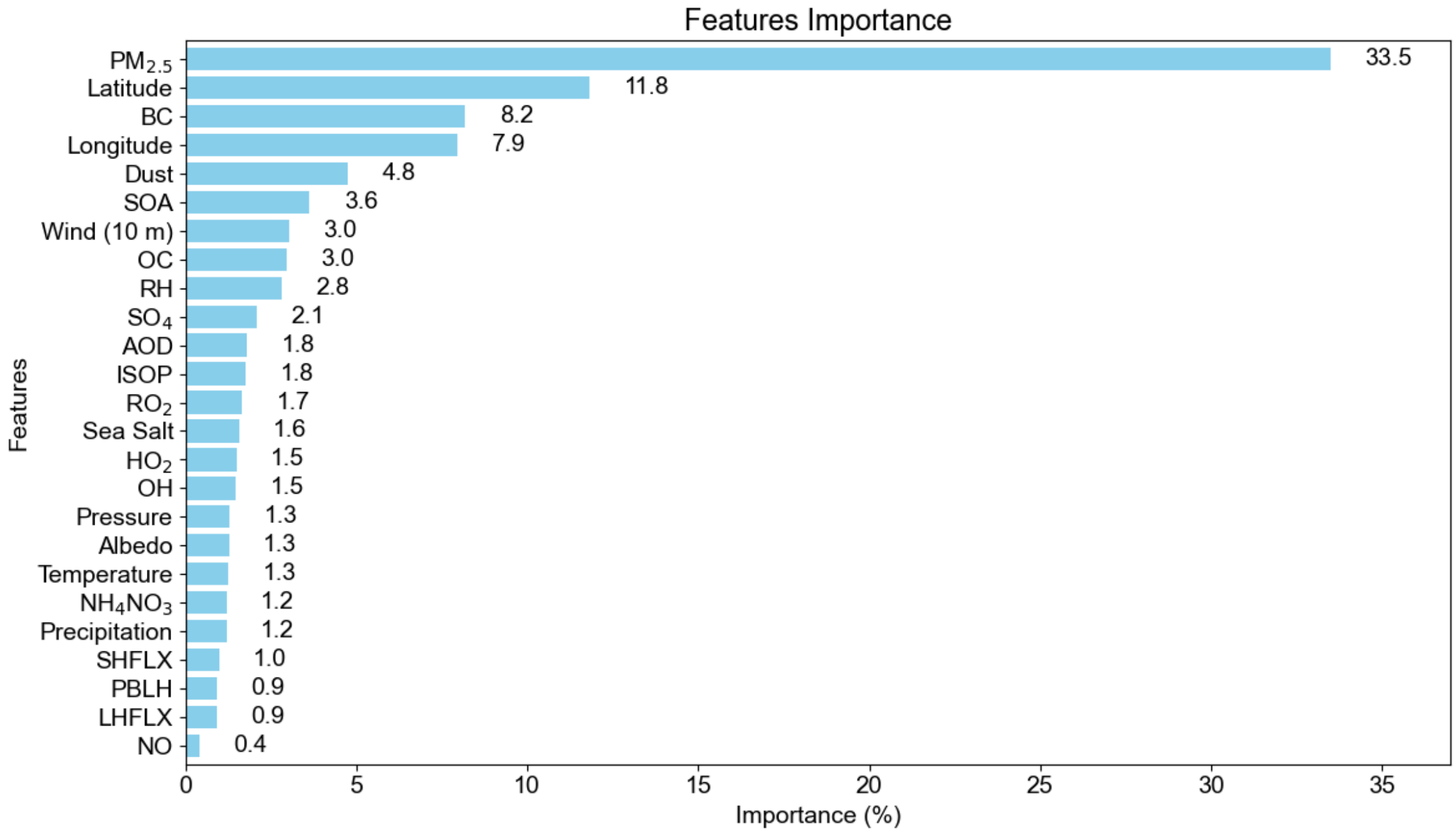

Figure S1. Feature importance for $PM_{2.5}$ concentrations. All the feature variables are obtained from the CESM simulations. Detailed information, including full name and units of all feature variables, is provided in Table S2.

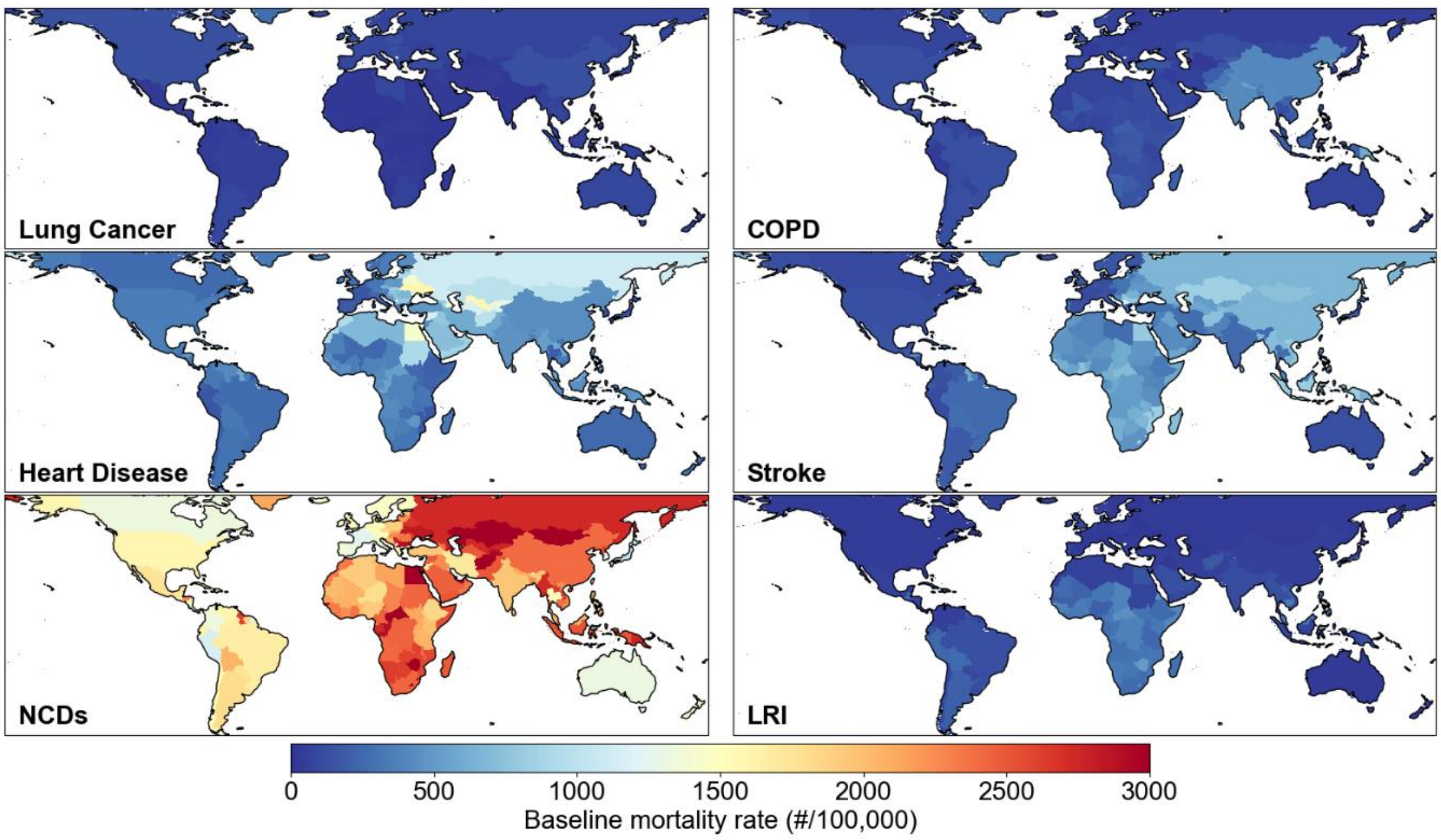

Figure S2. Baseline mortality rate for different causes of mortality for 2010 obtained from the Global Burden of Diseases (GBD: https://vizhub.healthdata.org/gbd-results/). These are averaged for 12 age-groups (25-29, 30-34, 35-39, 40-44, 45-49, 50-54, 55-59, 60-64, 65-69, 70-74, 75-79, and 80+).

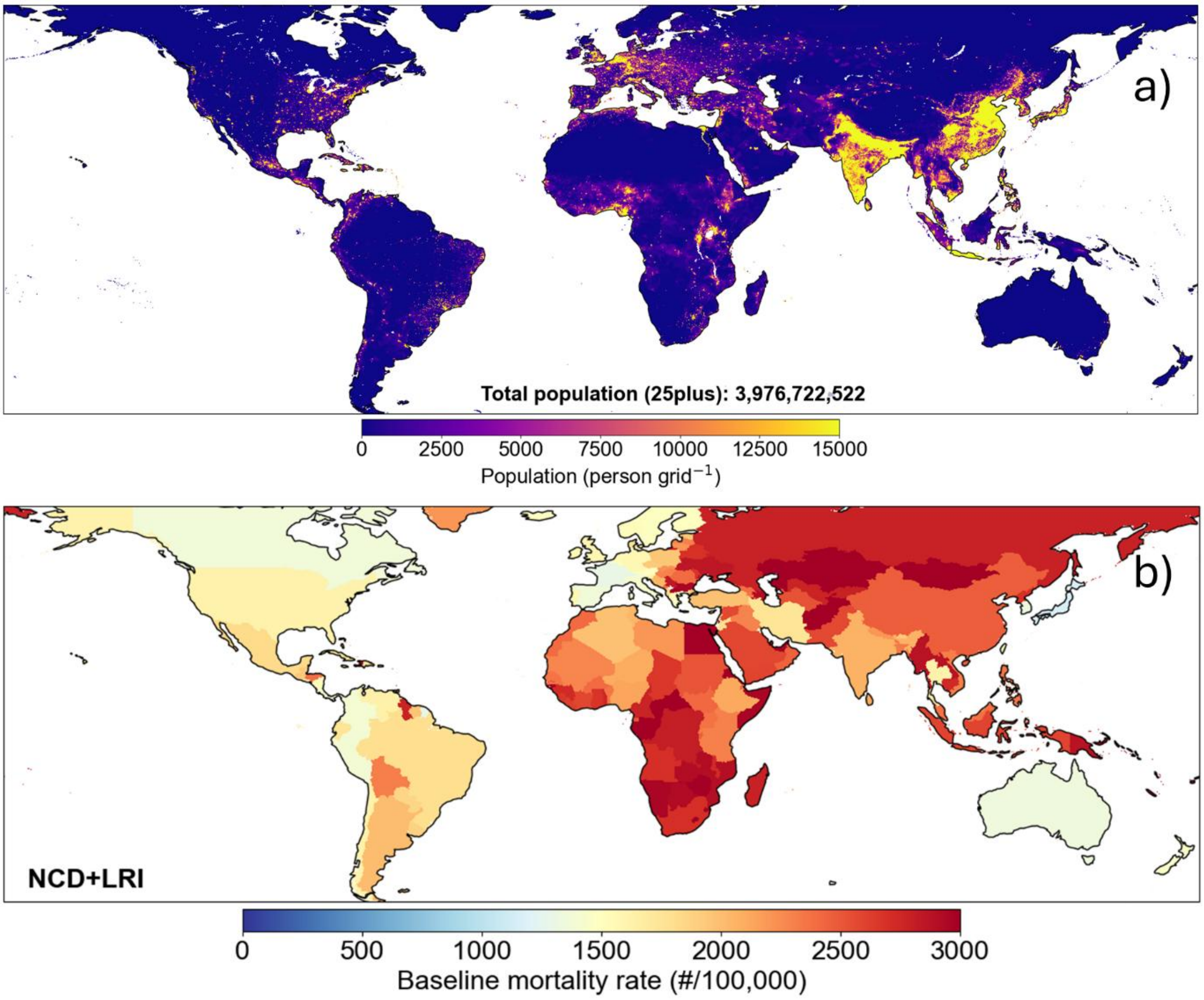

Figure S3. Baseline (2010) information of (a) population density for specific age group (25-84 years) from NASA Earth Data Centre, (b) baseline mortality rate for NCD+LRI obtained from Global Burden of Diseases (GBD).

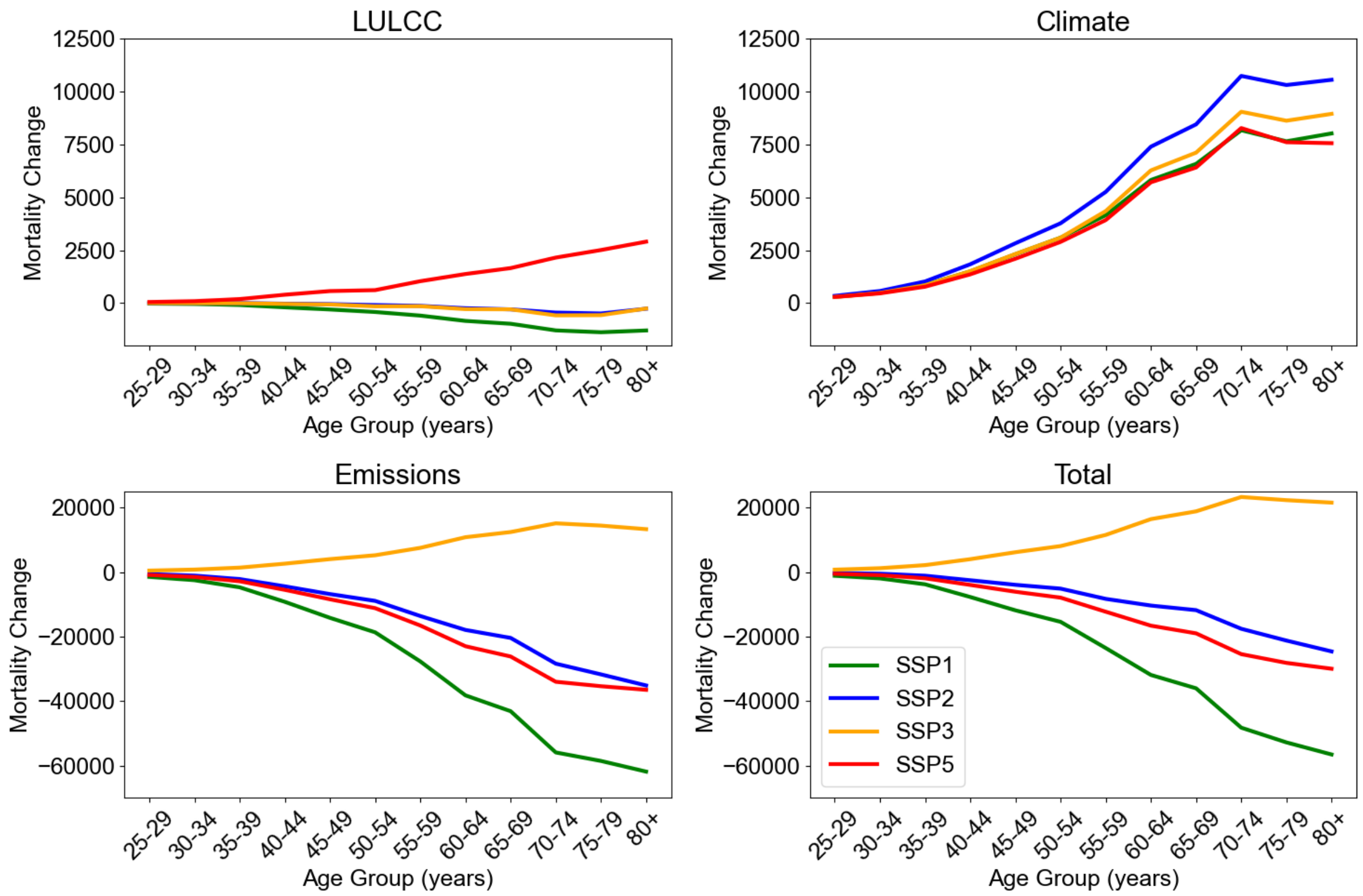

Figure S4. Age-specific mortality changes due to Strokes for four SSPs driven by land use (LULCC), climate, emissions, and their combined effects (total).

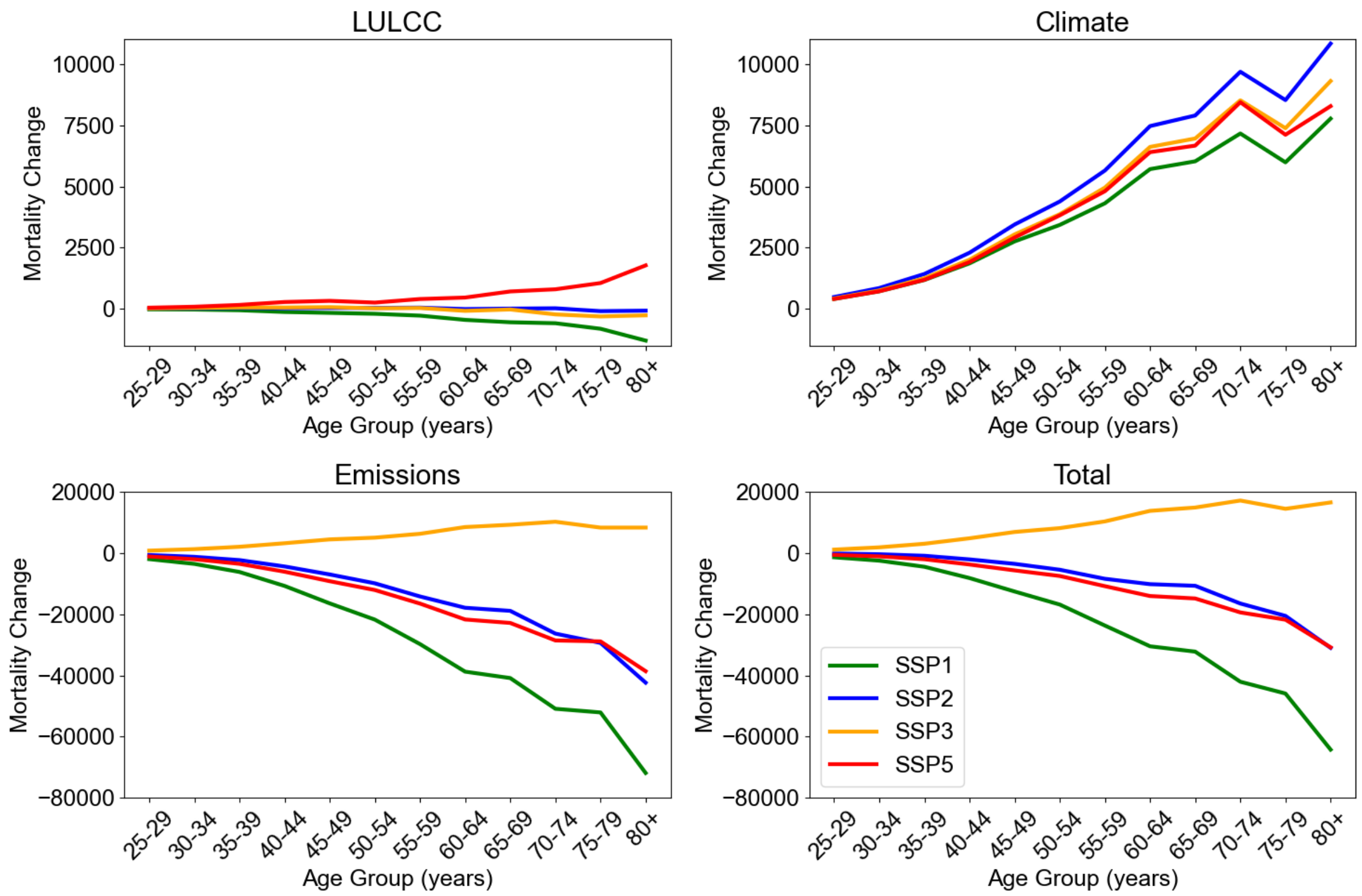

Figure S5. Same as Figure S4 but for IHD

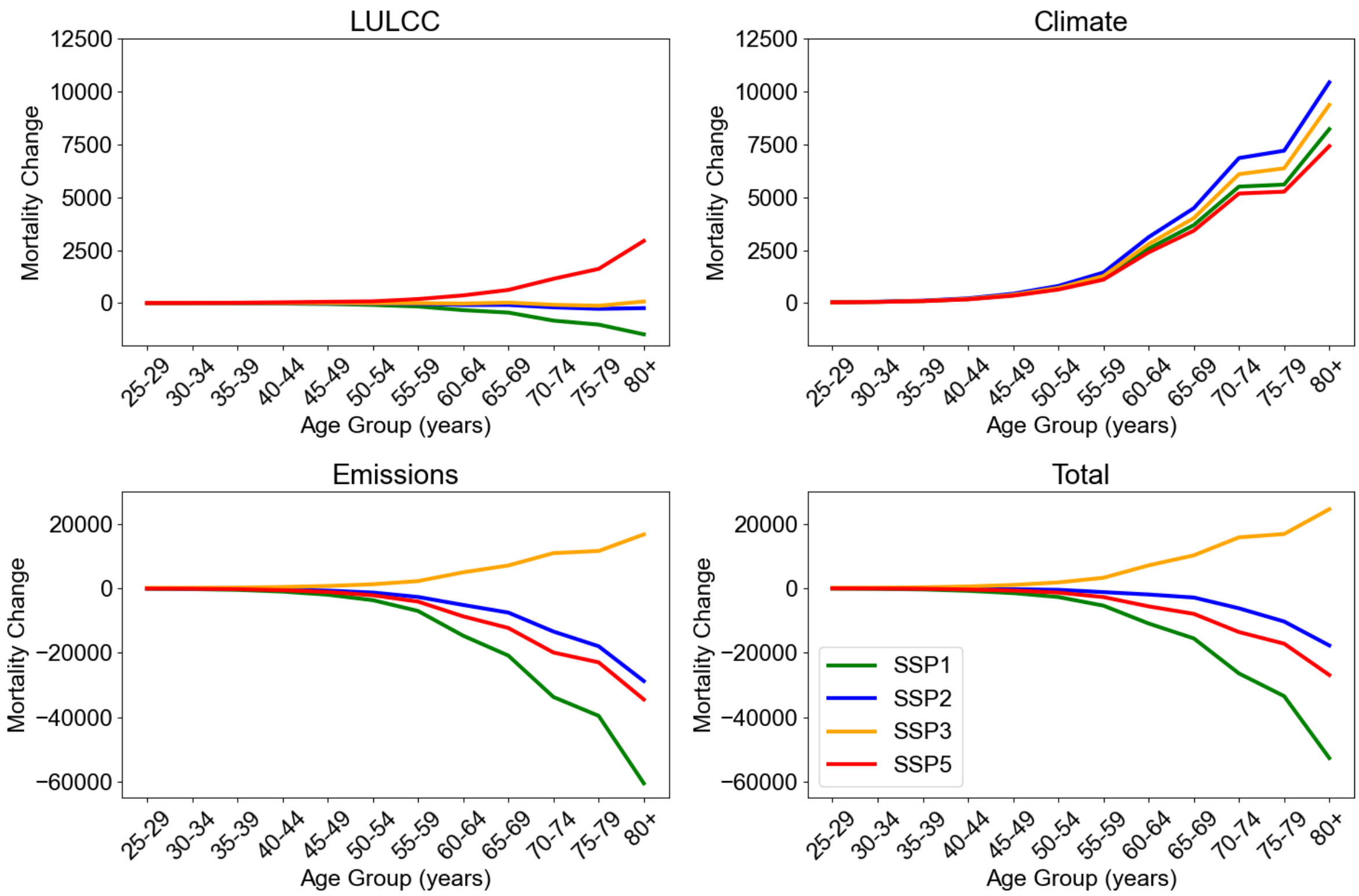

Figure S6. Same as Figure S4 but for COPD

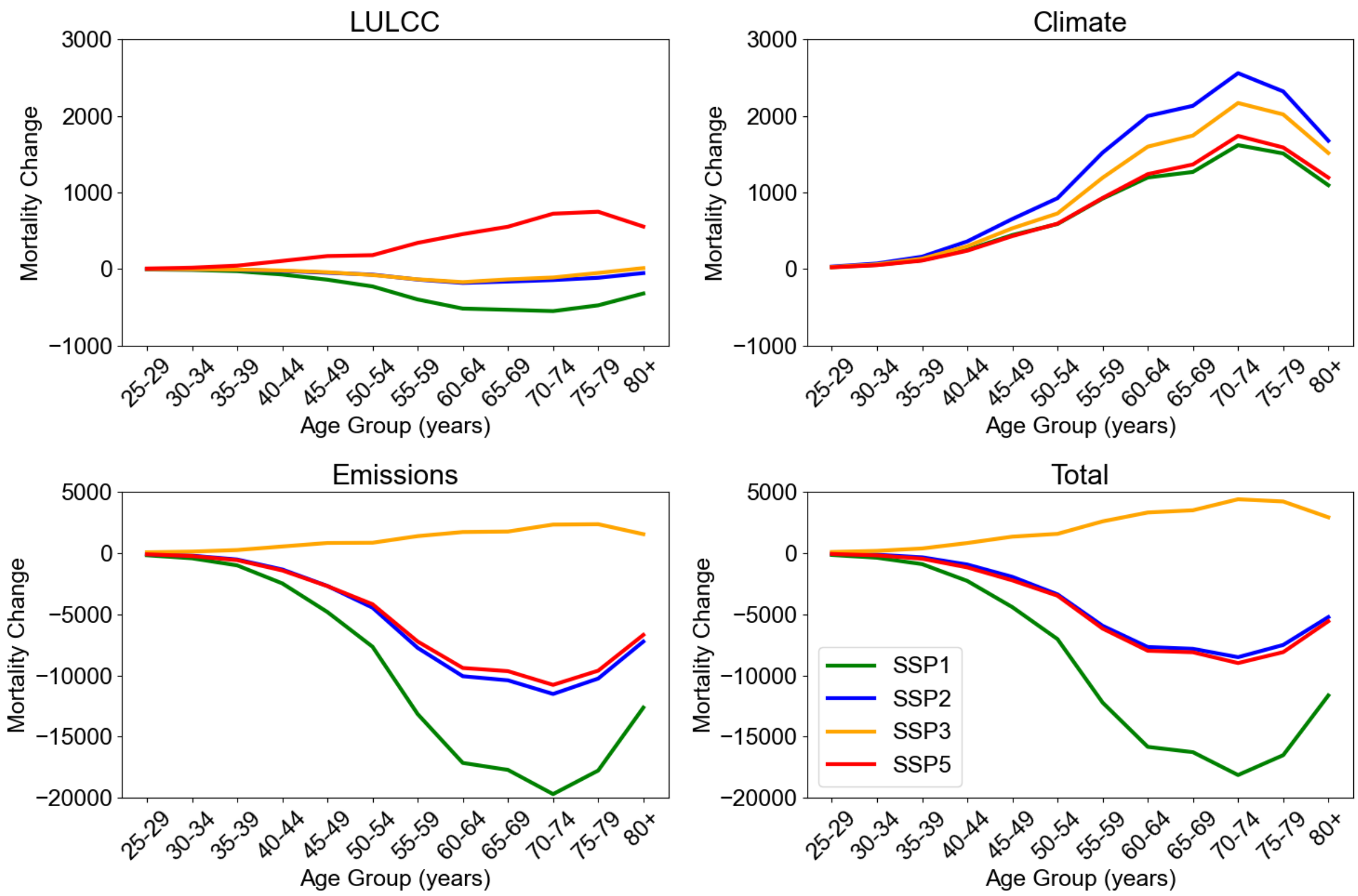

Figure S7. Same as Figure S4 but for Lung Cancer

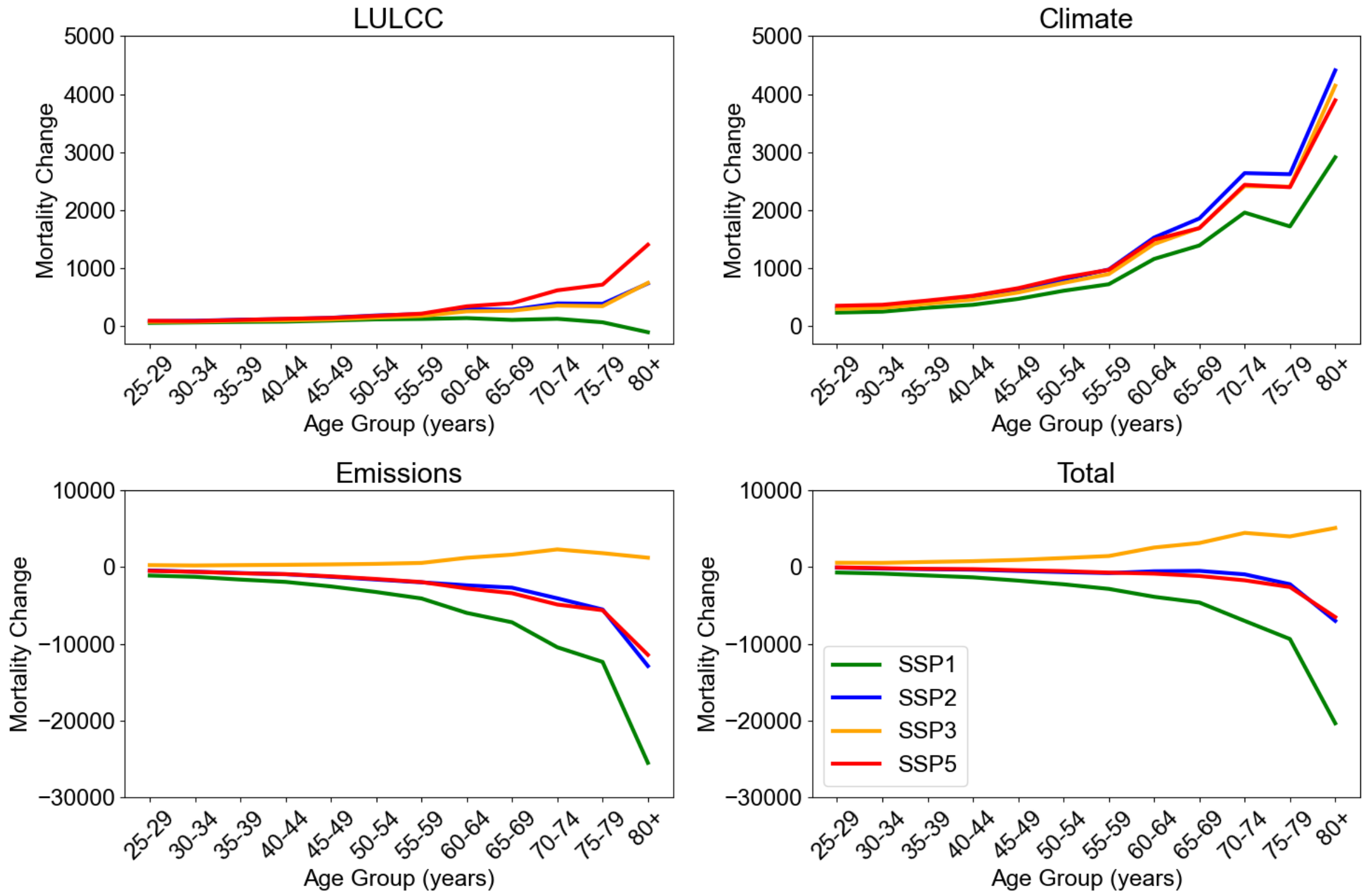

Figure S8. Same as Figure S4 but for LRI

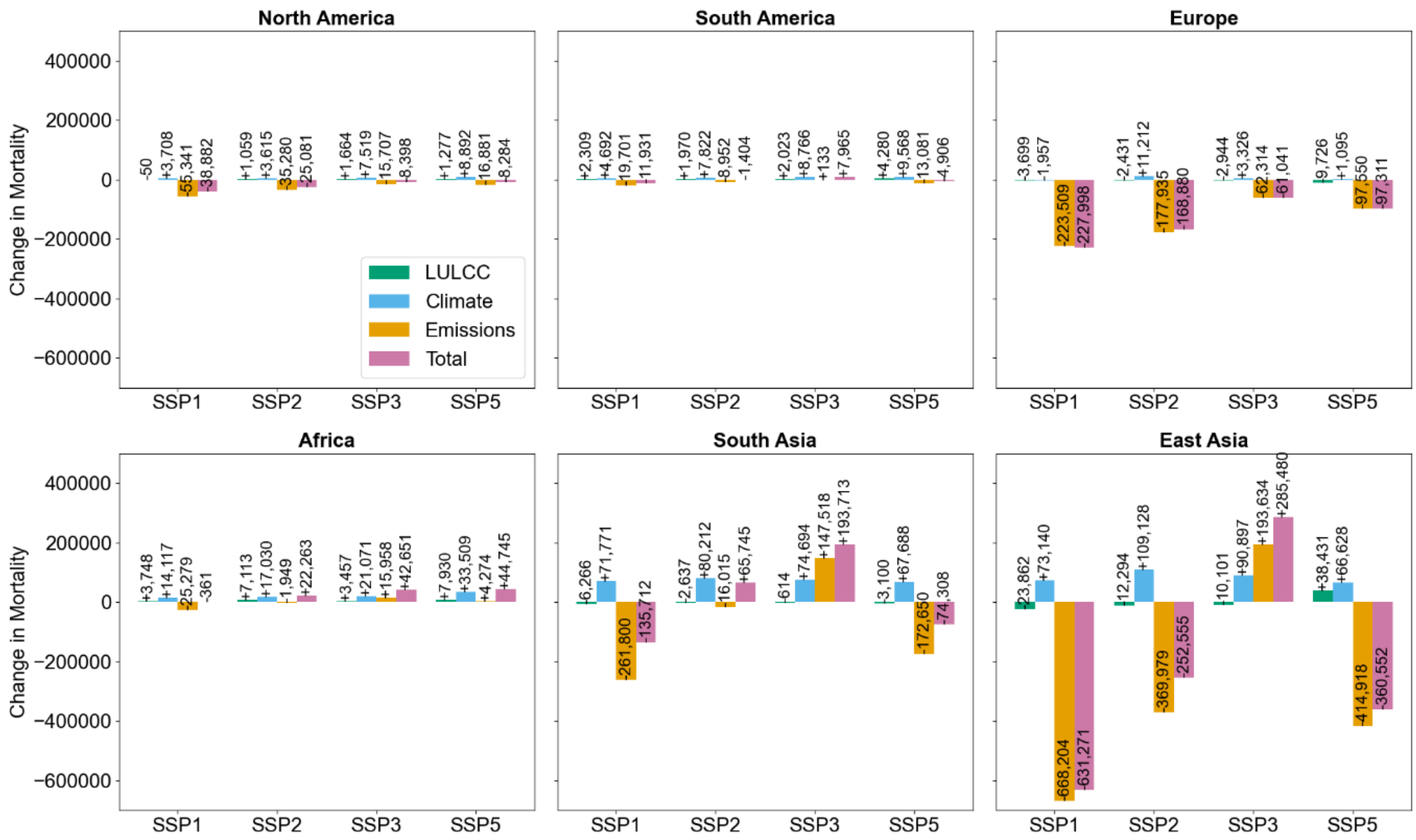

Figure S9. $PM_{2.5}$ induced mortality difference (NCD+LRI; absolute) for various regions under different SSP scenarios and different forcing factors.

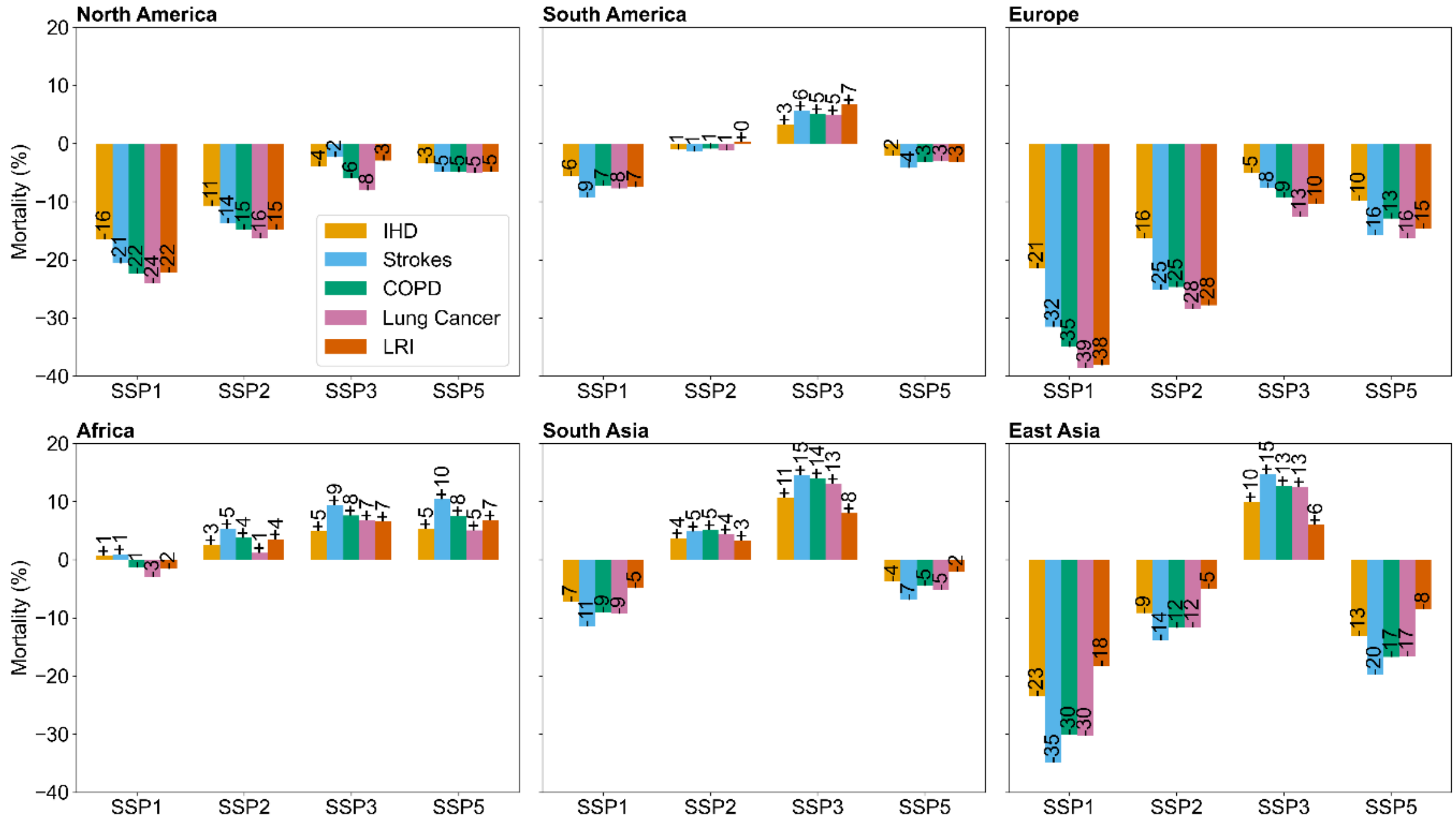

Figure S10. Disease-specific mortality difference (%) due to total (combined) forcing across different regions (North America, South America, Europe, Africa, South Asia, and East Asia) under Shared Socio-economic Pathways (SSPs: SSP1, SSP2, SSP3, SSP5).

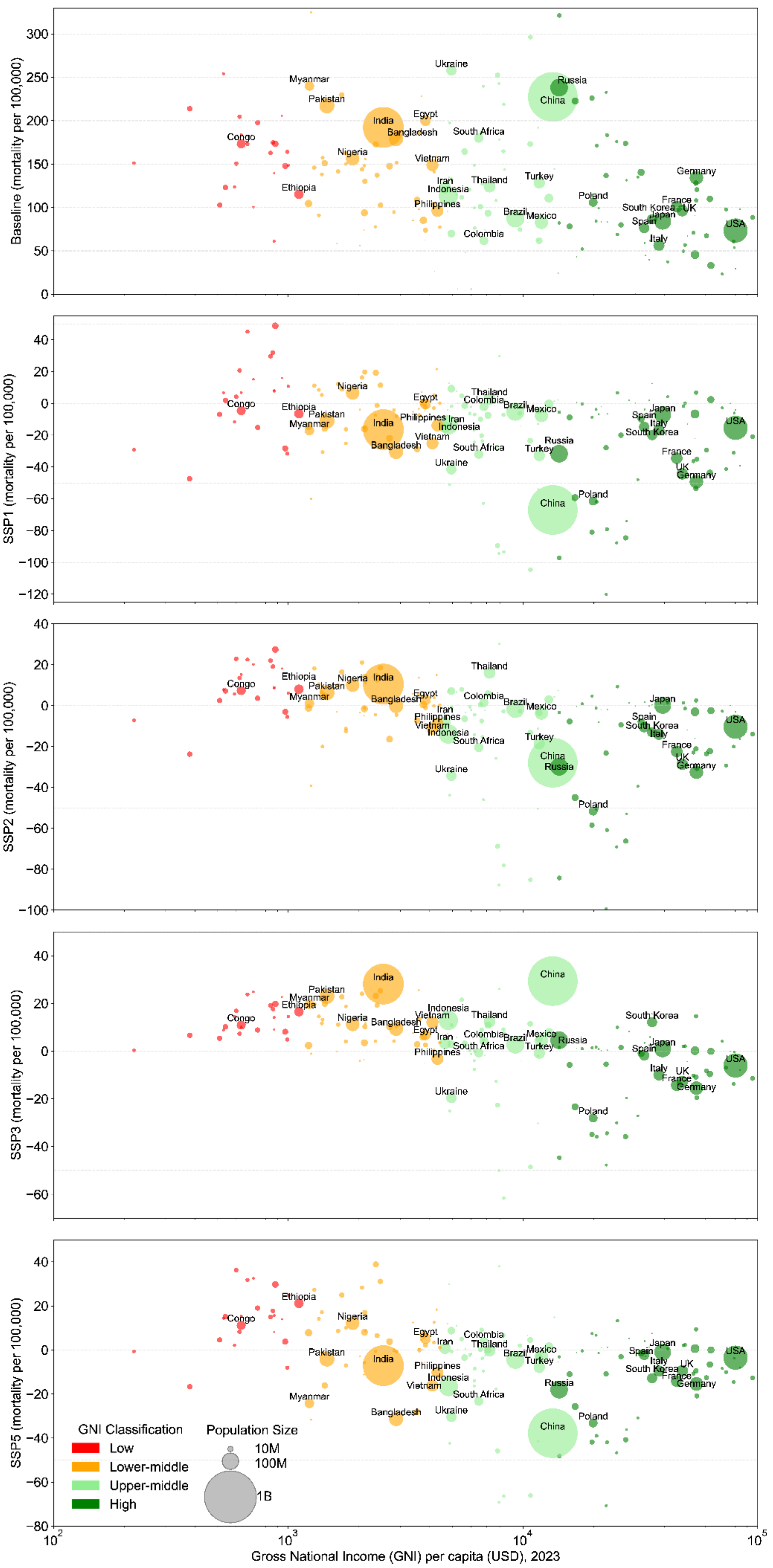

Baseline (mortality per 100,000)
SSP1 (mortality per 100,000)
SSP2 (mortality per 100,000)
SSP3 (mortality per 100,000)
SSP5 (mortality per 100,000)
Gross National Income (GNI) per capita (USD), 2023
GNI Classification
Low
Lower-middle
Upper-middle
High
Population Size
10M
100M
1B
India
China
Russia
USA
Germany
Japan
UK
France
Italy
Spain
South Korea
Poland
Turkey
Mexico
Brazil
Thailand
Colombia
South Africa
Ukraine
Iran
Indonesia
Philippines
Vietnam
Egypt
Bangladesh
Nigeria
Pakistan
Myanmar
Ethiopia
Congo

Figure S11. Baseline and future differences in $PM_{2.5}$-induced mortality under combined forcing (per 100,000) vs. gross national income (GNI) per capita for 2023. Bubble sizes indicate population, and colors represent income classifications (in USD), highlighting disparities in mortality across socioeconomic groups (low: ≤\$1,135, lower-middle: \$1,136–4,465, upper-middle: \$4,466–13,845, and high: >\$13,845). Only countries with top 30 population size (age 25+) are shown with names.
